# Feeding ecology and reproductive biology of small coastal sharks in Malaysian waters

**DOI:** 10.7717/peerj.15849

**Published:** 2023-08-21

**Authors:** Kean Chong Lim, Amy Yee-Hui Then, Kar-Hoe Loh

**Affiliations:** 1 Institute of Ocean and Earth Science, Universiti Malaya, Kuala Lumpur, Malaysia; 2 Institute for Advance Studies, Universiti Malaya, Kuala Lumpur, Malaysia; 3 Institute of Biological Science, Faculty of Science, Universiti Malaya, Kuala Lumpur, Malaysia

**Keywords:** Diet overlap, Resource partitioning, Ontogenetic, Size at maturity

## Abstract

Small coastal demersal sharks form a major proportion of the sharks landed in Malaysia. However, little is known about their feeding ecology and reproduction. This study sought to elucidate the dietary patterns, role of ontogeny in prey consumption, and reproductive biology of four dominant small demersal shark species in Malaysian waters: the Hasselt’s bamboo shark, *Chiloscyllium hasseltii*; brownbanded bamboo shark, *C. punctatum*; spadenose shark, *Scoliodon laticaudus*; and Pacific spadenose shark, *S. macrorhynchos*. Dietary analyses revealed a high overlap in prey taxa consumed; clear resource partitioning among co-occurring species based on the percentage Prey-specific Index of Relative Importance (%PSIRI), with higher fish %PSIRI for *Chiloscyllium hasseltii*, higher cephalopod %PSIRI for *C. punctatum*, and higher crustacean %PSIRI for both *Scoliodon* species; and an ontogenetic diet shift, seen through changes in prey size. Based on the examination of reproductive organs, the results showed larger sizes at maturity for males compared to females for all four species; no obvious reproductive cycles, based on hepatosomatic and gonadosomatic indices for all species; female bias in the sex ratio of the embryos of *Scoliodon* species; and increased reproductive output (number of eggs or embryos and size of eggs) with larger female size for *C. hasseltii* and *Scoliodon* species. The partitioning of food resources minimizes direct competition for food and supports coexistence within shared coastal habitats. The reproductive strategies of these small coastal sharks appear to be favorable for supporting short-term population productivity; although a reduction in fishing pressure, especially from bottom trawlers, is essential for the long-term sustainable use of these sharks.

## Introduction

Sharks play an important role in the top-down control and maintenance of healthy marine ecosystems (Ferretti et al., 2010; Heithaus, Wirsing & Dill, 2012). However, decadal studies suggest that many shark populations worldwide are experiencing a rapid decline owing to increased fishing efforts (Stevens et al., 2000; Schindler et al., 2002; Myers & Worm, 2005; Ferretti et al., 2008). The fishing of small coastal sharks is thought to be more sustainable than that of their larger, oceanic counterparts owing to a combination of lower fishing pressure, higher fecundity, and faster growth (García, Lucifora & Myers, 2008; Dulvy et al., 2014). However, fundamental information on the general biology and ecology of small coastal sharks is limited compared to that of the larger charismatic species (Huveneers, Ebert & Dudley, 2015; Troudet et al., 2017). These knowledge gaps are particularly problematic because small sharks are relatively abundant in tropical, multispecies fisheries that lack sustainable management (Cochrane, 2002; Arai & Azri, 2019; Booth et al., 2021; Lim et al., 2021).

A key knowledge gap is an understanding of the coexistence mechanisms of sympatric species which are influenced by several abiotic and biotic processes operating at local scales (Valladares et al., 2015). Resource partitioning is a fundamental biotic process that reduces competition for shared food resources, thus shaping the hierarchical dynamics of species and allowing the coexistence of multiple species in a single habitat (Navia, Mejía-Falla & Giraldo, 2007; Treloar, Laurenson & Stevens, 2007; Ruocco & Lucifora, 2016). This process is mainly influenced by a combination of the hunting abilities of predators and spatiotemporal prey dynamics (Laptikhovsky, Arkhipkin & Henderson, 2001; Dicken et al., 2017; Kousteni, Karachle & Megalofonou, 2017; Sánchez-Hernández, Gabler & Amundsen, 2017). The ability to hunt or forage is partially affected by changes in predator size and the competition between predator species that share similar resources. Spatiotemporal prey dynamics can be observed in dietary variations between sites and time of year, owing to changes in prey species diversity, abundance, and distribution (Devadoss, 1989; Saïdi et al., 2009; Torres-Rojas et al., 2015; Kousteni, Karachle & Megalofonou, 2017). These factors require further examination for poorly studied, small coastal sharks.

Understanding the reproductive dynamics in maintaining healthy populations (Coppock & Dziwenka, 2017) is another key knowledge gap for small coastal sharks. Sharks are generally considered highly susceptible to overfishing because of their life history strategies such as low fecundity, slow growth, and late maturity (García, Lucifora & Myers, 2008; Dulvy et al., 2014). However, sharks are known to exhibit a wide range of reproductive patterns (Conrath & Musick, 2012), and these traits may affect resilience to fishing in varying degrees across different species. A review by Cortés (2000) showed that female sharks, especially viviparous species, tend to mature at larger sizes than males. This maturation schedule may be an adaptation to the greater energy demands of females (Goodwin, Dulvy & Reynolds, 2002), although exceptions have been observed in some small sharks such as *Loxodon macrorhinus* (Gutteridge et al., 2013) and *S. macrorhynchos* (Zhao et al., 2022). Therefore, species-specific studies of reproductive patterns are essential to better evaluate the population health and fishery management of threatened but poorly studied sharks.

The bamboo sharks, *Chiloscyllium hasseltii* (Bleeker, 1852) and *C. punctatum* (Müller & Henle, 1838), and the spadenose sharks, *Scoliodon laticaudus* (Müller & Henle, 1838) and *S. macrorhynchos* (Bleeker, 1852), are small demersal sharks commonly found in the coastal waters of various Southeast Asian countries, including Malaysia (Lim et al., 2021; Lim et al., 2022). *C. punctatum* and both *Scoliodon* species are classified as Near Threatened under the International Union for Conservation of Nature and Natural Resources (IUCN) Red List of Threatened Species, whereas *C. hasseltii* is classified as Endangered (Dudgeon, Bennett & Kyne, 2016; Rigby et al., 2020; VanderWright et al., 2020; Dulvy et al., 2021). The IUCN status of *C. punctatum* in Southeast Asia may be uplisted to Vulnerable in the near future owing to high fishing pressure in the region (Dudgeon, Bennett & Kyne, 2016). In Malaysia, these species are mainly caught by bottom trawlers, collectively making up 10–88% of the total number of sharks landed (DOFM, 2006). All four species reportedly use similar habitats of sandy, muddy, and rocky substrates at depths of 85 m and up to 50 m for *Chiloscyllium* and *Scoliodon* species, respectively (Dudgeon, Bennett & Kyne, 2016; Rigby et al., 2020; VanderWright et al., 2020; Dulvy et al., 2021). The two *Scoliodon* species exhibited clear spatial segregation within Malaysian waters (Lim et al., 2022); however, they were found to co-occur individually with both *Chiloscyllium* species. The feeding ecology and coexistence mechanisms of these sympatric shark species in Malaysian waters remain poorly understood.

Limited dietary studies indicate that the four shark species in this study show some degree of specialization in their feeding. *C. hasseltii* from the east coast of Peninsular Malaysia show a preference for shrimp and fish (Nur-Farhana et al., 2013), whereas *C. punctatum* from Australia prefer crabs and fish (Gauthier et al., 2019). However, factors influencing the diet composition of these two *Chiloscyllium* species have not yet been examined. Studies have shown that *S. laticaudus* from Pakistan and India consume fish, crustaceans, and cephalopods; and the prey composition varies temporally and between sexes (Fofandi, Zala & Koya, 2013; Osmany, Manzoor & Zohra, 2018). The diet of *S. laticaudus* has also been shown to shift ontogenetically from slow-moving crustaceans, mainly prawns, to fast-moving fish (Devadoss, 1989). To the best of our knowledge, no dietary studies on *S. macrorhynchos* have been conducted.

Existing reproductive studies of small sharks suggest that females mature to larger sizes than males. This is observed for *C. punctatum* from Indonesia (Fahmi et al., 2021a) and *S. laticaudus* from the west coast of Peninsular Malaysia and India (Teshima, Ahmad & Mizue, 1978; Devadoss, 1979; Sen et al., 2018), but not for *S. macrorhynchos* in the northern South China Sea (Zhao et al., 2022). *C. punctatum* (Fahmi et al., 2021a), *S. laticaudus* (Sen et al., 2018), and *S. macrorhynchos* (Zhao et al., 2022) also appear to reproduce actively throughout the year, although different peak seasons were identified. Viviparous *Scoliodon* species show a positive correlation between female size and litter size, and a single female may carry up to 20 embryos in a single gestation period lasting 5–6 months (Teshima, Ahmad & Mizue, 1978; Devadoss, 1979; Sen et al., 2018; Zhao et al., 2022). To date, there have been no reproductive studies on the four shark species in Malaysian waters, and none are globally available for *C. hasseltii*.

The present study aimed to address these knowledge gaps by examining the diet composition and reproductive biology of these four common small shark species found in Malaysian waters. We were particularly interested in examining the role of body size on diet and reproductive output. Our results focus on the resource-partitioning strategies adopted by these co-occurring demersal sharks. Collectively, these findings provide fundamental information for sustainable fishery management plans for these and other demersal resources.

## Materials and Methods

### Field sampling

All field sampling was approved by the Research Council of Universiti Malaya (um.tnc1/606/stu2015 and um.tnc1/606/stu2016). The collection permits and sampling protocol for specimens from Sabah were approved by the Sabah Biodiversity Council (Access License Reference No: JKM/MBS.1000-2/2 JLD.9 (21–23) and Transfer License Reference No: JKM/MBS. 1000-2/3 JLD.4 (18)). Both *C. hasseltii* and *C. punctatum* were the most abundant species landed by fisheries operating in the waters of both the east and west coasts of Peninsular Malaysia. *S. macrorhynchos* was the most common species caught in Sarawak waters, whereas *S. laticaudus* was ranked third in landings from the west coast of Peninsular Malaysia (DOFM, 2006).

**Figure 1:**
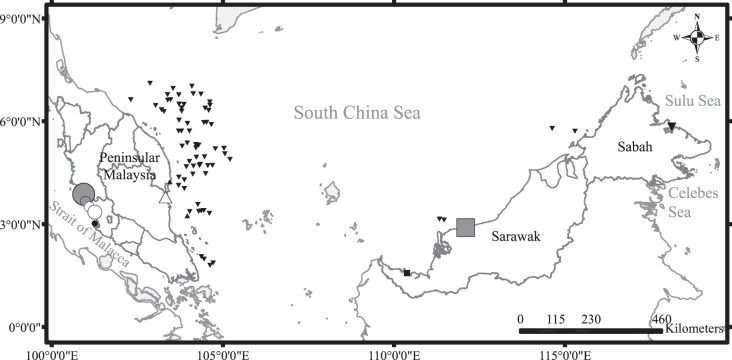
Sampling sites around Malaysia with symbols indicating diversity of species sampled (number of species and individuals per species). Black upward triangle, *C. hasseltii*; black downward triangle, *C. punctatum*; black circle, *S. laticaudus*; black square, *S. macrorhynchos*; grey circle, combination of *C. hasseltii*, *C. punctatum* and *S. laticaudus*; grey square, combination of *C. hasseltii, C. punctatum* and *S. macrorhynchos*; white circle, combination of *C. hasseltii* and *S. laticaudus*; white upward triangle, combination of *C. hasseltii* and *C. punctatum*. Size of symbols indicate sample size.

Dead specimens of all four species were sampled during survey trips to major landing sites and markets across Malaysia (Fig. 1) from May 2015 to October 2016. Monthly surveys on the central west coast of Peninsular Malaysia were conducted to characterize temporal changes in reproductive effort. Surveys in other areas were conducted at less regular intervals: two on the east coast of Peninsular Malaysia, three in Sarawak, and one in Sabah. Sharks were mainly caught by trawlers operating beyond 12 nautical miles, except for those sampled from Sarawak which were caught using both inshore gill nets and trawlers. The sharks were transported to the laboratory and frozen until further processing. Additional samples were obtained during research-based demersal trawl surveys of Malaysian South China Sea waters organized by the Department of Fisheries Malaysia (DOFM). Owing to logistic limitations, most of the samples from the demersal trawl surveys were assessed for maturity on-site but were not processed for dietary analysis.

### Laboratory analysis

All sharks were measured for total length, sex, and maturity stages. The classification of maturity stages, mature or immature, was based on both the external and internal reproductive organs (Lim, 2016). Mature males displayed elongated and calcified claspers with well-developed testes, showing clear formation of lobes; greater than 50% of the total size of the testes. Mature females had enlarged uteri, which in some cases were bearing eggs or embryos, and ovaries with well-developed ova. Immature sharks of both sexes had thread-like or partially developed gonads in addition to small and soft claspers in males and thin uteri in females. The liver (W_L_) and gonads (W_G_) were dissected and weighed to the nearest 0.01 g. For gravid females, the number and length of eggs in *Chiloscyllium* species, or the number of embryos and their sex in *Scoliodon* species, was recorded.

Stomachs were dissected from the anterior end (esophagus) to the posterior end (immediately after the pyloric sphincter) and preserved in 70% ethanol prior to fixation in 10% formalin. All prey items were identified to the lowest possible taxa and their numbers were recorded. Volumetric contributions of larger prey items were estimated using the water displacement method (Hyslop, 1980), whereas those of smaller prey items were estimated using gridded 1 ml Sedgewick rafter cells examined under a stereomicroscope.

### Data analysis

Diet composition was summarized according to species and maturity using percent volume (*%V*), percent number (*%N*), percent frequency of occurrence (*%F*), and percent Prey-Specific Index of Relative Importance (*%PSIRI*), as outlined by Brown et al. (2012).

${\rm\%} V = V_i V_{t}^{-1} \times 100{\rm\%}$

${\rm\%} N = N_i N_{t}^{-1} \times 100{\rm\%}$

${\rm\%}F = F_i n^{-1} \times 100{\rm\%}$

${\rm\%}PSIRI = 0.5 \,({\rm\%}V + {\rm\%}N)$

where *V*_*i*_ is the total volume of the prey item *i*, *V*_*t*_ is the total volume of all prey items, *N*_*i*_ is the total number of prey items *i*, *N*_*t*_ is the total number of prey item*s, F*_*i*_ is the number of stomachs containing prey item *i*, and *n* is the number of stomachs examined.

The *%PSIRI* was used instead of *%IRI* because the latter showed undesirable mathematical properties for describing prey diets (Brown et al., 2012). Two indices were used to evaluate the general niche breadth of the shark species: Levin’s index (*B*) and its standardized form (*B*_*A*_) and the adapted Berger-Parker dominance index (*D*) (Krebs, 1999; Levins, 1968).

$B = \left(\sum p_i^2\right)^{-1} \rm and$

$B_A = (B \,-\,1) (N - 1)^{-1},$

where *p*_*i*_ is the proportion of each prey taxon consumed, and *N* is the total number of food categories recognized. The adapted Berger-Parker index, based on *%PSIRI*, directly refers to the proportion of the most important prey items for each species. The standardized form of Levin’s index gives a value between 0 and 1; where values less than 0.6 refer to specialist feeders, whereas values greater than 0.6 refer to generalist feeders (Labropoulou & Eleftheriou, 1997). The adapted Berger-Parker index (*D*) provides values range from 0.01–1. Values closer to 0.01 indicate generalist feeders, whereas values closer to 1 indicate specialist feeders.

A graphical approach using prey-specific abundance (*P*_*iN*_) or volume (*P*_*iV*_) *vs %F* was applied to further describe species-specific feeding strategies by combining the five most important prey items of each studied species (Amundsen, Gabler & Staldvik, 1996).

$P_{iN} = \left(\sum N_i /\sum N_{ti}\right) \times\rm 100, or$

$P_{iV} = \left(\sum V_i /\sum V_{ti}\right) \times\rm 100,$

where *N*_*i*_ or *V*_*i*_ is the number or volume of prey *i*, and *N*_*ti*_ or *V*_*ti*_ is the total number or volume of predator stomachs that contained prey *i*. Prey item position in the vertical axis space represents the feeding strategy, either specialist (prey item position in the top half of the graph) or generalist (prey item position in the bottom half of the graph). Starting from upper right quadrant in a clockwise manner, the four quadrants with the center at 50%F and 50%*P*_*iNorV*_ represent dominant; high within-phenotype component, commonly found but in low quantity; rare; and high between-phenotype component, rarely found but in high quantity.

Dietary data were randomly pooled for every two to eight individuals using the average for *V*, *N*, and *F* and recalculated for *%PSIRI* according to species and maturity; subsequently referred to as dietary samples. This pooling strategy was employed to reduce intraspecific variability in diet (Pardo et al., 2015; Platell & Potter, 2001). Similarity matrices for the dietary samples were constructed using the Bray-Curtis dissimilarity coefficient after square root transformation. The matrices were then analyzed using PERMANOVA with an additional Monte Carlo test to compare the variation in diet among species and maturity stages. The results based on the Monte Carlo test were used when the number of unique permutations was less than 20, which was insufficient to make a significant statistical inference (Anderson, 2005). The maturity stages were pooled when there was no significant difference identified in the PERMANOVA test.

Similarity percentage analysis (SIMPER) identified the key prey items responsible for dietary differences among species. Species-specific cumulative prey curves were constructed to determine whether the collected samples sufficiently represented the diet of the species (Cortés, 1997) and were plotted as an average of 999 curves based on random orders of non-empty stomachs examined (Clarke & Gorley, 2006). The number of prey taxa ingested individually (*N*_*pi*_) was compared among species using the Kruskal-Wallis one-way analysis of variance (ANOVA), followed by *post-hoc* pairwise comparisons. To examine ontogenetic diet shifts, linear regression analysis between the volume of the largest prey item found in each stomach (*PV*_*max*_) for both overall and dominant prey taxa and shark size, as total length, was performed after natural logarithmic transformation (Lim et al., 2019).

The maturity of all the specimens was coded binomially (0 = immature and 1 = mature). The size at first maturity (TL_1_) was determined from the smallest mature individuals recorded. The total length at which 50% of the individuals attained maturity (TL_50_) was derived using logistic regression, according to the method by White, Platell & Potter (2001). The proportion of mature individual, P_mature_, was calculated using P_mature_ = 1/[1 + e^−(*a*+*b*TL)^], where *a* and *b* are constants. These constants were estimated for each species and sex, using a general linear/nonlinear model module with binomial distribution. TL_50_ was then calculated using the equation TL_50_ = −*a*/*b*.

Only mature sharks collected from the monthly surveys were used to determine the hepato-somatic index (HSI = 100 W_L_/W_T_) and gonadosomatic index (GSI = 100 W_G_/W_T_). Boxplots of monthly HSI and GSI were generated to observe seasonal variations in liver and gonad size in both males and females. The relationship between the reproductive effort (number of embryos or eggs, length of embryos, or eggs) and female size was determined.

PRIMER v6 (Clarke & Gorley, 2006) was used for SIMPER, PERMANOVA, and cumulative prey curves; and Statistica 8.0 software (StatSoft Inc, 1997) was used for ANOVA, linear, and logistic regressions.

## Results

### Prey diversity and feeding patterns

**Figure 2:**
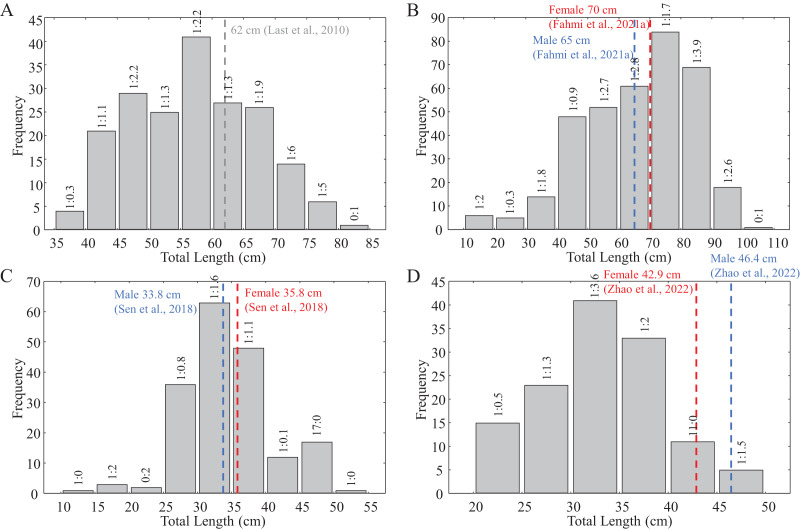
Size frequency distribution of the collected shark samples for reproductive biology. (A) *C. hasseltii*, (B) *C. punctatum*, (C) *S. laticaudus*, (D) *S. macrorhynchos*. Dashed lines indicate maturity size for each species from available literature: Red, female; Blue, male; Grey, unspecified.

The stomach contents of 129 *C. hasseltii*, 137 *C. punctatum*, 145 *S. laticaudus*, and 57 *S. macrorhynchos* were examined. Of these, 44 stomachs were found to be empty (Table 1). The size-frequency distribution of the examined specimens encompassed juveniles, subadults, and adults of wide-ranging sizes and maturity levels (Table S1 and Fig. 2). Forty-five prey taxa including 23 families of teleost fish, 11 crustaceans, five cephalopods, four other invertebrates, ‘others’, and unknown items were identified (Table 2). The category of ‘others’ included items that were rarely found and potentially ingested accidentally such as fishing net fragment, cephalopod eggs, stingray spine, and tapeworm; while the ‘unknown’ category referred to partially digested items that could not be identified. Cumulative prey curves indicated that the sample sizes were sufficient (slope < 0.1) for *C. punctatum* and *S. laticaudus* and approximately sufficient for both *C. hasseltii* (0.12) and *S. macrorhynchos* (0.11) (Fig. S1).

**Table 1:** Total stomachs examined according to species, location and their size range.

Species	Location	Total stomachs	Empty stomachs	Size range (cm)	
				Min	Max
*C. hasseltii*	WP	128	4	37.8	80.6
	Sarawak	1	0	61.0	61.0
	All	129	4	37.8	80.6
*C. punctatum*	WP	124	13	30.5	94.8
	EP	1	0	14.2	14.2
	Borneo	12	1	54.0	87.8
	All	137	14	14.2	94.8
*S. laticaudus*	WP	145	22	15.8	51.3
*S. macrorhynchos*	Sarawak	57	4	21.7	45.2

**Note:**

WP, west coast of Peninsular Malaysia; EP, east coast of Peninsular Malaysia; Borneo, Sarawak and Sabah.

**Table 2:** Prey-specific index of relative importance (*%PSIRI*) for each prey taxa according to elasmobranch species (Chas—*C. hasseltii*, Cpun—*C. punctatum*, Slat—*S. laticaudus*, Smac—*S. macrorhynchos*) and their respective niche breadth values.

Prey taxa	Abbrev.	Common name	Chas	Cpun	Slat	Smac
Teleosts			47.4	5.6	30.1	30.2
Actinopterygii	Actin	Bony fish	**12.2**	3.2	**17.1**	**13.3**
Anguiliformes	Angui	Eel	1.8	1.4		
Ambassidae	Ambas	Glass fish	0.7		2.0	
Ammodytidae	Ammod	Sand lances	1.3			
Apogonidae	Apogo	Cardinal fish	0.2			
Bregmacerotidae	Bregm	Codlets	0.3		3.1	
Carangidae	Caran	Trevally	2.4			
Clupeidae	Clupe	Shads			1.0	1.7
Cynoglossidae	Cynog	Tongue sole	4.9			**7.9**
Engraulidae	Engra	Anchovy	2.3	0.4	4.7	5.1
Leiognathidae	Leiog	Ponyfish	1.8	0.5		
Mugilidae	Mugil	Mullet	0.5			
Mullidae	Mulli	Goat fish	5.3			
Nemipteridae	Nemip	Sea bream	0.3			
Pinguipedidae	Pingu	Sand perch	0.3			
Platycephalidae	Platy	Flathead	0.2			
Sciaenidae	Sciae	Croaker	1.7		0.4	
Serranidae	Serra	Grouper	0.2			
Siganidae	Sigan	Rabbit fish	0.2		1.1	1.4
Soleidae	Solei	Flatfish	0.5			
Synodontidae	Synod	Lizard fish	**9.7**			
Tetraodontidae	Tetra	Pufferfish				0.8
Trichiuridae	Trich	Cutlass fish	0.8		0.7	
Crustaceans			11.4	13.6	49.8	37.6
Unknown shrimp	Unshr		0.8	2.5	5.4	5.0
Penaeidae	Penae	Penaeid shrimp	**6.6**		**11.3**	**15.6**
Caridea	Carid	Caridean shrimp	0.9	1.3	**10.2**	1.5
Sergestidae	Serge	Sergestid shrimp	0.3	0.9	**10.8**	1.3
Stomatopoda	Stoma	Mantis shrimp	0.1	0.3	8.9	**7.4**
Unknown crab	Uncra		1.8	**5.3**	2.2	3.7
Portunidae	Portu	Swimming crab	0.3	2.6	0.6	2.5
Calappidae	Calap	Box crab	0.6	0.7		
Paguroidae	Pagur	Hermit crab	0.2		0.2	
Calanoida	Calan	Copepod			0.1	
Ostracoda	Ostra	Ostracod			0.2	0.6
Cephalopods			27.4	71.0	16.5	26.7
Unknown cephalopod	Cepha		4.9	**17.9**	**14.4**	2.7
Teuthida	Teuth	Squid	**17.2**	**27.0**	2.1	**24.0**
Sepiolidae	Sepio	Bobtail squid	2.0	**5.5**		
Sepiida	Sepii	Cuttlefish	0.3	1.9		
Octopoda	Octop	Octopus	3.0	**18.8**		
Other invertebrates			13.6	7.3	1.5	0.6
Bivalvia	Bival	Bivalve		0.2		
Polychaete	Polyc	Bristle worm	**6.3**	3.6	0.4	
Nematoda	Nemat	Round worm	4.5	2.7	0.5	0.6
Isopoda	Isopo	Isopod	2.8	0.6	0.6	
Others	Oth			0.2	0.5	3.2
Unknown	Unk		0.2	2.3	1.7	1.6
Niche breadth index						
B			13.06	6.59	9.93	8.20
B_A_			0.32	0.25	0.37	0.35
D			0.17	0.27	0.17	0.24

**Notes:**

Top five prey taxa for each species are shown in bold.

B, Levine’s index; B_A_, Levine’s standardize index; D, Berger-Parker dominance index.

Individual sharks consumed between one and seven types of prey taxa, each with a mean *N*_*pi*_ of 2.0 (Table S2). *C. hasseltii* showed the highest mean *N*_*pi*_ (2.8), whereas *S. macrorhynchos* showed the lowest mean *N*_*pi*_ (1.4). *N*_*pi*_ was not normally distributed, and comparisons of *N*_*pi*_ among shark species were, therefore, based on a *post-hoc* analysis using the Kruskal-Wallis test (Table S2). *C. hasseltii* had a significantly higher *N*_*pi*_ than the other species.

Seven prey taxa were found to contribute more than 10%PSIRI to the diet of at least one shark species (Table 2). These included Actinopterygii (maximum %PSIRI = 17.1), Penaeidae (%PSIRI = 15.6), Caridea (%PSIRI = 10.2), Sergestidae (%PSIRI = 10.8), unknown cephalopod (%PSIRI = 17.9), Teuthida (%PSIRI = 27.0), and Octopoda (%PSIRI = 18.8). The sharks primarily consumed teleosts (%PSIRI = 3.7–55.2), shrimp (%PSIRI = 0–53.6), and cephalopods (%PSIRI = 10.1–75.3); supplemented with other invertebrates (%PSIRI = 0–24.3) and crabs (%PSIRI = 0–16.0) (Fig. S2). The contribution of smaller crustaceans, such as hermit crabs, calanoid copepods, and ostracods, was found to be negligible (maximum %PSIRI = 1.4). The diet composition of *C. punctatum* was distinctly different from that of the other species and included larger quantities of cephalopods; whereas *C. hasseltii* and *S. laticaudus* consumed higher quantities of teleosts and shrimp, respectively (Fig. S2). Equal diet contributions from teleosts, shrimp, and cephalopods were observed for *S. macrorhynchos*. Significant variations in diet among species were confirmed by the PERMANOVA test on dietary samples (main test Pseudo-F = 10.036, *P*-value < 0.01; *P*-value < 0.01 for all pairwise comparisons).

Among the prey items, SIMPER analysis showed that Teuthida was the most important contributor to diet dissimilarity among the shark species, followed by Actinopterygii and Penaeidae (Table S3). For sub-indices of PSIRI, the main contributor to dissimilarity by volume was Teuthida, followed by Penaeidae and Octopoda/Actinopterygii; the main contributor to dissimilarity by number was unknown cephalopods, followed by Actinopterygii and Sergestidae. Intra- and interspecific variations in diet were considered to be large, based on the low similarity index within (<40%, except *C. punctatum*) and between species (<30%) (Table S4).

Based on standardized Levin’s index values (<0.6), all four species were categorized as specialist feeders (Table 2). In contrast, the Berger-Parker index values were closer to 0.1, indicating that all four species exhibited a generalist feeding behavior. The graphical approach using prey-specific abundance or volume indicated generalist feeding for both *C. hasseltii* and *S. laticaudus* but specialist feeding for *C. punctatum* and *S. macrorhynchos* (Fig. S3). Prey specialization in *C. punctatum* was observed for Sergestidae, Stomatopoda, and the cephalopods Teuthidae and Octopoda (Fig. S3B). *S. macrorhynchos* specialized in predating on nine prey taxa, including Actinopterygii (unidentified fish), Cynoglossidae, Engraulidae, Penaeidae, Caridea, Sergestidae, Stomatopoda, crabs, and Teuthida (Fig. S3D).

There was no obvious change in the prey items with increasing body size for any shark species (Fig. S2). A strong positive correlation between prey volume and shark size was observed for both *Chiloscyllium* species; however, this correlation was weakly positive for both *Scoliodon* species (Fig. S4). The diet contribution of teleosts, particularly the lizardfish Synondotidae, for *C. hasseltii* increased with body size; whereas that of other invertebrates decreased. For *C. punctatum*, limited changes in prey contributions were observed (Fig. S2). The diet of juveniles included crustaceans Caridea, Sergestidae, and Stomatopoda in their diet; however, these prey taxa were replaced with the addition of Anguiliformes, Leiognathidae, Calappidae, Sepiidae, and Bivalvia in adult stomachs. As *S. laticaudus* matured, an increase in its consumption of crustaceans, especially Sergestidae and Stomatopoda, was observed (Fig. S2). In addition, there was a slight decrease in the consumption of teleosts and cephalopods. This pattern differed from that of *S. macrorhynchos* where a greater reliance on teleost prey and less reliance on shrimp and cephalopods was observed after maturation (Fig. S2).

### Reproductive biology

**Figure 3:**
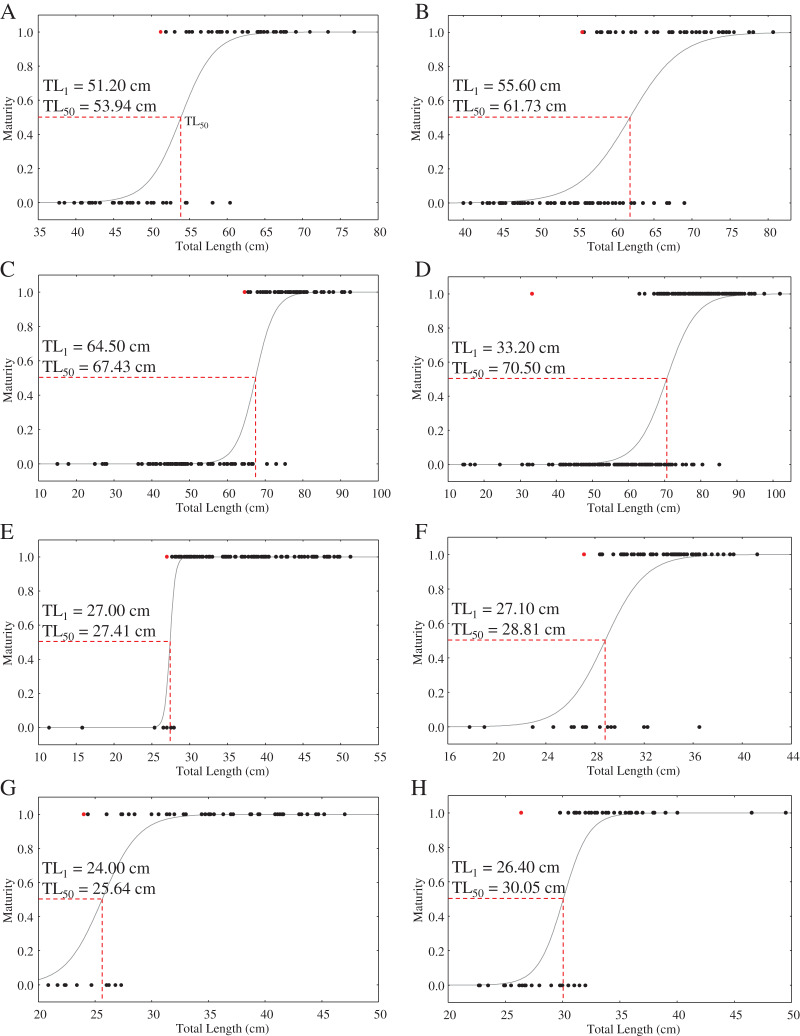
Maturity logistic regression for all sharks according to sex (female—left column, male—right column). (A and B) *C. hasseltii*, (C and D) *C. punctatum*, (E and F) *S. laticaudus*, (G and H) *S. macrorhynchos*.

With the exception of a particularly small mature *C. punctatum* male, most species showed a size at first maturity (TL_1_) that was relatively similar to the estimated size at 50% maturity (TL_50_) (Fig. 3). Fitted logistic regression showed that in all species, females matured at smaller sizes than the males (Fig. 3). The estimated TL_50_ for female *C. hasseltii* was 53.9 cm (95% confidence interval (CI) [51.9–56.1 cm]), 61.7 cm (95% CI [59.8–63.8 cm]) for male *C. hasseltii*, 67.4 cm (95% CI [65.2–69.8 cm]) for female *C. punctatum*, 70.5 cm (95% CI [68.7–72.3 cm]) for male *C. punctatum*, 27.4 cm (95% CI [26.8–28.0 cm]) for female *S. laticaudus*, 28.8 cm (95% CI [27.3–29.9 cm]) for male *S. laticaudus*, 25.6 cm (95% CI [27.3–27.3 cm]) for female *S. macrorhynchos*, and 30.1 cm (95% CI [28.6–31.1 cm]) for male *S. macrorhynchos*.

Based on monthly sampling, the adult HSI and GSI for all species fluctuated considerably throughout the year (Fig. S5). The combined ranges of HSI and GSI for the sharks from locations other than the west coast of Peninsular Malaysia were as follows: mean HSI 3.7% (range 3.2–4.2%) and mean GSI 3.8% (range 3.7–3.9%) for female *C. hasseltii*; mean HSI 3.7% (2.4–5.4%) and mean GSI 3.5% (1.6–6.2%) for female *C. punctatum*; mean HSI 4.0% (3.3–5.0%) and mean GSI 0.9% (0.6–1.2%) for male *C. punctatum*; mean HSI 3.5% (1.7–5.3%) and mean GSI 0.5% (0.3–0.8%) for female *S. macrorhynchos*; and mean HSI 3.1% (2.1–4.7%) and mean GSI 1.0% (0.5–1.6%) for male *S. macrorhynchos*. No visible temporal trend was observed for the HSI of the three species and sexes examined, except for male *C. hasseltii* which showed peaks in November 2015 and October 2016. A seasonal cycle of GSI was observed in female *C. hasseltii*, male *C. punctatum*, and female *S. laticaudus*; but not in the opposite sexes of these species.

**Figure 4:**
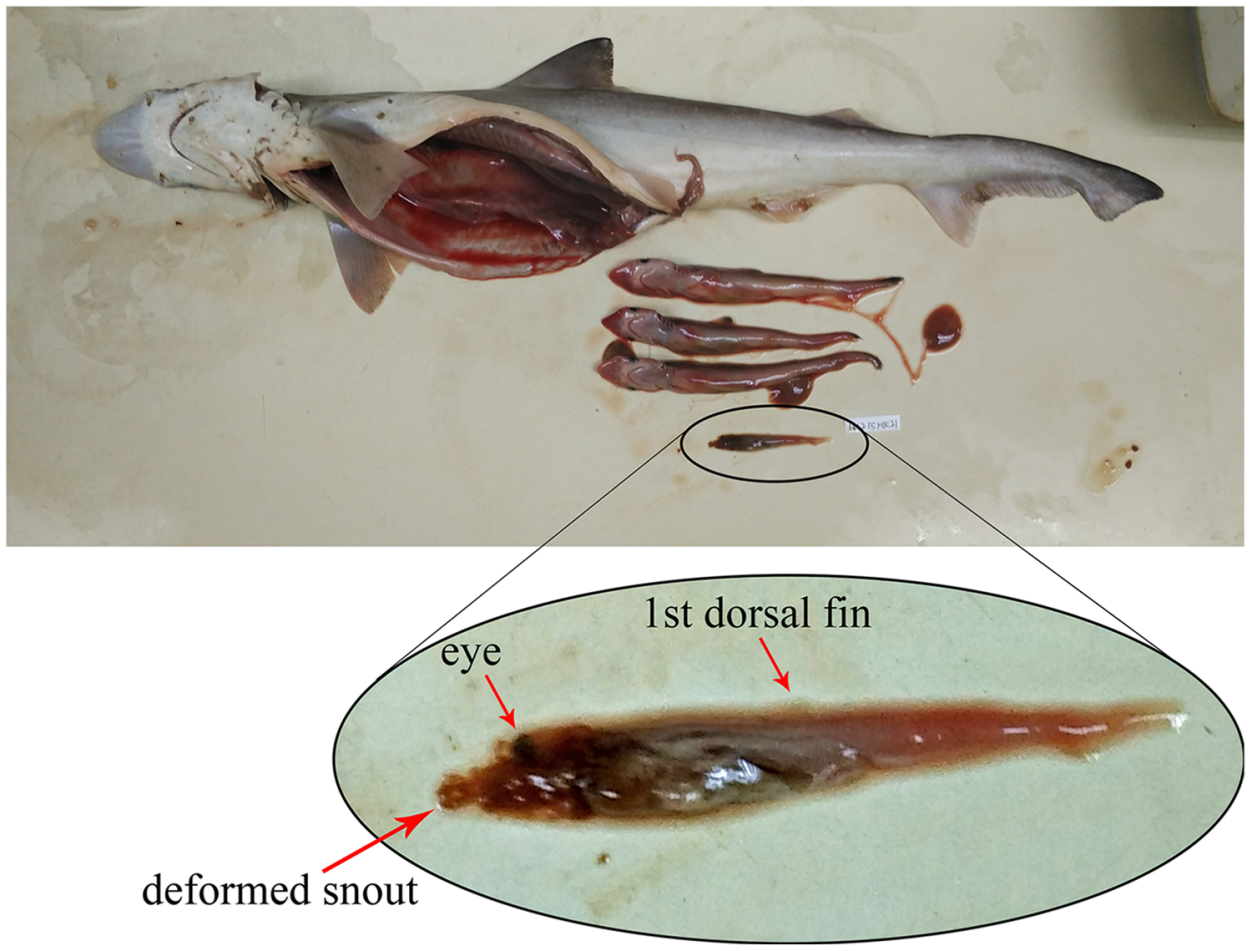
Observation of either congenital deformity or intrauterine cannibalism in *Scoliodon macrorhynchos*. One underdeveloped embryo with deformed head.

Based on the 80 gravid female sharks (24 *C. hasseltii*, 10 *C. punctatum*, 27 *S. laticaudus*, and 19 *S. macrorhynchos*), the uterine fecundity was determined as 1–4 egg cases (mean = 2) in *C. hasseltii*, 1–2 egg cases (mean = 2) in *C. punctatum*, 1–12 embryos (mean = 5) in *S. laticaudus*, and 1–14 embryos (mean = 5) in *S. macrorhynchos*. The female to male sex ratio for embryos were 1:0.52 and 1:0.68 for *S. laticaudus* and *S. macrorhynchos* respectively. The number of eggs or embryos was positively correlated with female size for all species, except for *C. punctatum* (Fig. S6, left column). Egg size positively correlated with female size in *C. hasseltii* (Fig. S6B). In both *Scoliodon* species, large variations in embryo size (TL) were seen within one single adult female *Scoliodon*. This variation ranged from 0–3.0 cm (mean = 0.5 cm) for *S. laticaudus* and 0–6.7 cm (mean = 1.0 cm) for *S. macrorhynchos*. The observation of one headless and underdeveloped embryo, along with three other well-developed embryos, was recorded in one female *S. macrorhynchos*; suggesting either a congenital deformity or intrauterine cannibalism (Fig. 4).

## Discussion

Based on data collected across Malaysia, we found clear evidence of resource partitioning among the four co-occurring abundant, small coastal demersal sharks. Ontogenetic shifts in diets were primarily observed in the selection of larger-sized prey rather than in switching to different prey taxa. Females of all the species matured to smaller sizes than males, which is a less common reproductive strategy among sharks. Periodicity during reproduction was not discernible in any of the species. Larger adult *Scoliodon* females with a TL greater than 43 cm were associated with higher numbers of embryos (≥8). The possibility of intrauterine cannibalism, which has been reported to occur mainly in larger sharks such as sand tiger sharks, requires further investigation. These findings are one of the first for Malaysia’s small shark populations and have implications for fisheries management of these sharks.

### Dietary patterns and resource partitioning

All four sharks exhibited generalist feeding patterns and exploited a high diversity of prey taxa, comprising both demersal- and water column-associated species. Among the 20 fish taxa, the benthic lizardfish, Synodontidae, was the most important fish prey; and the pelagic-associated squid, Teuthida, was the most important cephalopod prey in the diets of the four sharks. The types and contributions of consumed prey reflect the diversity in the feeding behaviors of these sharks. This is demonstrated by the large number of demersal organisms in their diets, including eels and polychaetes, which burrow on the seafloor. Sharks are opportunistic feeders, feeding on diverse prey located in close proximity and, thereby, reducing the energy spent and increasing net energy intake (Heller, 1980). The diversity of prey taxa seen in our study is consistent with previous studies on *S. laticaudus* that showed predation on 16 to 33 prey taxa (Wang et al., 1996; Fofandi, Zala & Koya, 2013; Osmany, Manzoor & Zohra, 2018) and studies on other coastal demersal shark species, such as *C. plagiosum, Heterodontus portusjacksoni, Mustelus canis, M. henlei, M. lunulatus*, and *M. mustelus* that showed consumption of 13 to 77 identified prey taxa (Jardas et al., 2007; Navia, Mejía-Falla & Giraldo, 2007; Saïdi et al., 2009; Sommerville et al., 2011; Woodland, Secor & Wedge, 2011; Amariles, Navia & Giraldo, 2017; Liu et al., 2020).

Diet composition for a given species differs between locations owing to the variation in both prey and predator species diversity (Heithaus & Vaudo, 2012; Lim et al., 2019). Prey species diversity directly affects prey choice. Our findings showed that fish account for almost half of the diet of *C. hasseltii*, followed by cephalopods. This differs from an earlier dietary study of the same species in Malaysia which found a high proportion of shrimp (Nur-Farhana et al., 2013). We found that *C. punctatum* appeared to prefer cephalopods. This differs from observations of *C. punctatum* in Australia that consume mainly crabs and fish (Gauthier et al., 2019). These spatial differences probably reflect variations in the types of prey available locally. The seasonality of prey availability is another important consideration that could not be examined in our study because of the limited sample size.

Predator species diversity shapes interspecific competition for available food resources. Predators of similar trophic levels may partition themselves by habitat (O’Shea et al., 2013), resources (Navia, Mejía-Falla & Giraldo, 2007; Treloar, Laurenson & Stevens, 2007; Ruocco & Lucifora, 2016), or even feeding times (Cortés, 1997; Bangley & Rulifson, 2017) to avoid the need to directly compete with each other unless resources are abundant (Laptikhovsky, Arkhipkin & Henderson, 2001). Our diet analysis provides evidence of resource partitioning among co-occurring sharks, indicating that available food resources may be limited to a small, shared home range area for these sharks. Reliance on wide-ranging prey taxa allows these small sharks to switch their targets when faced with competition from larger sharks and non-shark predators. Complete prey carcasses, both intact and fragmented, were seen in most of the stomach contents, with no visible signs of body parts being torn apart; suggesting that the sharks did not exhibit food-stealing behavior associated with interference competition (kleptoparasitism) (Heithaus & Vaudo, 2012).

Generalist feeders have a competitive advantage over specialist feeders for thriving in degraded habitats (McKinney & Lockwood, 1999; Layman & Allgeier, 2012). Most shark samples in this study were obtained by bottom trawling, which is highly destructive to bottom habitats and may necessitate the exploitation of multiple food resources, including pelagic ones. A characteristic shared by these four small species is short-distance swimming. For example, *Chiloscyllium* species are inactive swimmers and are commonly found in groups among crevices (Compagno, 2001; Wilga & Lauder, 2004). Niche breadth indices indicated a continuum range of specialist to generalist feeding patterns, suggesting individual specialization of prey taxa within a generalist species, as seen in other predators (Woo et al., 2008; Scholz et al., 2020). Collective evidence using *N*_*pi*_, total prey taxa, *B*_*A*_, *D*, and *%PSIRI* indicated that *C. hasseltii* exhibited the most generalist feeding patterns, followed by *S. laticaudus*, *S. macrorhynchos*, and *C. punctatum*. These dietary patterns are probably important for ensuring the survival of these sharks in highly degraded environments that experience high fishing pressure (Lim et al., 2021).

Ontogenetic diet shifts are common in sharks (Bornatowski et al., 2014; Dicken et al., 2017; Gračan et al., 2017; Kousteni, Karachle & Megalofonou, 2017; Liu et al., 2020). These shifts with increasing shark size are consistent with the optimum foraging theory owing to increased energy requirements. Additionally, ontogenetic diet shifts also benefit smaller, younger sharks by reducing competition of shared resources, thus increasing overall population survival. Our study showed that the ontogenetic diet shift observed in the four sharks was mediated by the selection of larger-sized, similar prey rather than switching to different prey types. This strategy makes sense energetically for small sharks with a limited swimming range and may be facilitated by enhanced spatial resolving power with increased size, which in turn improves the predatory capability to track larger, more nutrient-rich prey; as seen in *C. punctatum* (Harahush, Hart & Collin, 2014).

### Reproductive biology

The size at maturity (TL_50_) of both *Chiloscyllium* species in Malaysian waters was similar to that reported by Last et al. (2010). Some spatial variation in size at maturity was observed for *C. punctatum*, with females in Malaysian waters having a smaller mature size (TL_50_ = 67.4 cm) compared to those from neighboring Indonesia (70.5 cm; Fahmi et al., 2021a). In contrast, male *C. punctatum* in Malaysian waters matured at a larger size (TL_50_ = 70.5 cm) than that reported in Indonesia (65.3 cm; Fahmi et al., 2021a). Regional variability in size at maturity, coupled with population genetic structuring (Lim et al., 2021; Fahmi et al., 2021b), supports localized reproductive studies to inform fisheries management at spatially relevant scales.

Sizes at maturity for *S. laticaudus* reported in the present study (TL_50_ = 27.4 cm for female, 28.8 cm for males) were smaller than previous records in the same area (TL_50_ = 32.4 cm for female, 33 cm for males; Teshima, Ahmad & Mizue (1978)), as well as those in India (TL_50_ = 35 cm for female, 33 cm for male; Devadoss (1979), Sen et al. (2018)). The lack of mature size estimates for *S. macrorhynchos* in Sarawak prevented similar comparisons. Compared to *Chiloscyllium* species, the meat of *Scoliodon* species fetches a higher market value, is used in local cuisines (K. C. Lim, A. Y. Then, 2015–2016, personal observations), and is much less likely to be discarded as bycatch. The earlier maturity schedule observed for *S. laticaudus* compared to previous studies probably reflects the high fishing pressure on sharks in the Malacca Strait waters. A reduction in the maturity size of exploited species is a common response to heavy fishing pressure (Stergiou, 2002; Stevens et al., 2000).

A meta-analysis reviewing reproductive strategies in sharks highlighted that females tend to mature at larger sizes than their male counterparts to improve the survival of the young through better nutrient support in their early development (Cortés, 2000). However, our study found the opposite pattern in all four shark species. This pattern is a strategy commonly adopted by small carcharhinids to maximize their lifetime reproductive output while coping with higher natural mortality rates than their larger counterparts (Gutteridge et al., 2013). Given the high fishing pressure experienced by demersal resources in Malaysia, including sharks; early female maturation may be a population-level response to high natural and fishing mortality rates (Azri & Arai, 2015; Arai & Azri, 2019).

We did not find clear seasonality in the reproductive cycles of any of the four sharks. The high variability in the monthly HSI and GSI data indicates that sharks are in various reproductive stages over time. Both gravid and non-gravid females were found in each sampling trip, and embryos of various sizes were detected each month (1.2–11.2 cm for *S. laticaudus* and 2.2–12.8 cm for *S. macrorhynchos*). The continuous breeding of these sharks is consistent with that of other species from tropical regions (White, 2007; Fahmi et al., 2021a). Without clear seasonality in the reproductive cycle, the gestation period of sharks could not be estimated. No near-term embryos were recorded in this study. This raises the concern of capture-induced parturition, such as premature birth or abortion, (Adams et al., 2018) and further studies using different sampling methodologies will be important to ascertain whether this is a problem for the *Scoliodon* species.

The uterine fecundity of these species was similar to the results of previous studies (Harahush, Fischer & Collin, 2007; Sen et al., 2018; Fahmi et al., 2021a; Zhao et al., 2022) and was higher for the viviparous *Scoliodon* species than for the oviparous *Chiloscyllium* species. According to Harahush, Fischer & Collin (2007) and Chen & Liu (2006), *Chiloscyllium* species lay one to two eggs every four to ten days. Considering the high reproductive frequency, a lower number of eggs per reproductive session allows for higher nutrient reserves for embryonic development. In contrast, *Scoliodon* species give birth to one to 20 embryos after 6 months (Sen et al., 2018; Zhao et al., 2022). This higher uterine fecundity is probably required to compensate for the relatively long gestation period. A positive correlation between fecundity and female size is common among sharks, mainly because of the increase in uterine space in larger individuals or species (Parsons et al., 2008). An exception to this can be seen in species such as the sand tiger shark, *Carcharias taurus*, which shows extreme uterine cannibalism such as adelphophagy or sibling eating. No previous study has documented uterine cannibalism in *Scoliodon* species, and our study suggests the possibility of this occurring in *S. macrorhynchos*. Although additional samples, especially those with near-term embryos, are necessary to verify this, we hypothesize that uterine cannibalism in *S. macrorhynchos* is associated with higher numbers of embryos and greater variability in their size, in the larger females.

### Limitations and future studies

The limited temporal sampling of dietary data in this study prevented the clarification of seasonality in shark prey consumption. Stomach content analysis alone provides a snapshot of individual diets but may not reflect what is being assimilated over a longer period of time. Stable isotope analysis of muscle tissue can provide additional insights into spatiotemporal patterns and ontogenetic shifts in the diet of these sharks (Rosende-Pereiro et al., 2020). Our sampling approach, through visits to fish landing sites and adjacent markets, limited the size range of sharks encountered to larger juvenile and adult individuals. Informal conversations with local fishing operators found that unmarketable bycatch may include smaller shark individuals that are usually discarded or sent directly to fish feed processing factories. Investigation of the diet of smaller-sized juveniles will provide clarity on diet preference during early life stages and any ontogenetic diet shifts. Future studies should also include sampling of the diversity and abundance of candidate prey to elucidate the role of prey selectivity in the shark diets.

## Conclusions

The four dominant small demersal shark species in Malaysian waters, *C. hasseltii*, *C. punctatum*, *S. laticaudus*, and *S. macrorhynchos*, exploit diverse prey taxa that occupy both demersal and pelagic environments but also exhibit clear resource partitioning in their diets. This reflects the possible limitations of available food resources within their small home ranges. The generalist feeding strategy employed reduces intra- and interspecific competition, thus conferring ecological resilience to these species that use degraded bottom habitats. The females of these small sharks, especially *Scoliodon* species, appear to mature earlier than in the previously observed. This reproductive strategy appears to be a response to high fishing pressure, but may be detrimental to long-term population survival owing to increased vulnerability to predation with smaller birth sizes (Cortés, 2000). Given the limitations of both the home range and dispersal abilities of these small coastal sharks; an overall reduction in fishing pressure, especially from bottom trawlers, is important to ensure their sustainability and that of other demersal resources.

## Supplemental Information

10.7717/peerj.15849/supp-1 Supplemental Information 1 Cumulative prey curve according to elasmobranch species. Grey upward triangle, *C. hasseltii*; black downward triangle, *C. punctatum*; empty circle, *S. laticaudus*; black square, *S. macrorhynchos*.

10.7717/peerj.15849/supp-2 Supplemental Information 2 Percentage contribution (based on *%PSIRI*) of six combined general groups compared among various elasmobranch species. Im, immature; Ma, mature.

10.7717/peerj.15849/supp-3 Supplemental Information 3 Scatterplots of prey specific abundance/volume of the most important prey items against their frequency of occurrence for three species of elasmobranch. (A) *C. hasseltii*, (B) *C. punctatum*, (C) *S. laticaudus*, and (D) *S. macrorhynchos*. Grey symbols, prey specific abundance; black symbols, prey specific volume.

10.7717/peerj.15849/supp-4 Supplemental Information 4 Scatterplot between prey volume (mm^3^) and predator size (total length in cm). (A) *Chiloscyllium hasseltii*, (B) *C. punctatum*, (C) *S. laticaudus*, and (D) *S. macrorhynchos*.

10.7717/peerj.15849/supp-5 Supplemental Information 5 Monthly Hepato-somatic index, HSI (left column) and Gonado-somatic index, GSI (right) from September 2015 to October 2016 for adult sharks of *C. hasseltii* (top row), *C. punctatum* (middle), *S. laticaudus* (bottom). Grey filled box, female (F); Unshaded box, male (M). Range and mean (in parenthesis) were stated on the lower left corner.

10.7717/peerj.15849/supp-6 Supplemental Information 6 Linear regression between number of eggs/embryos and total length of mother (A, C, E, G) and between eggs/embryos length and total length of mother (B, D, F, H) for: (A & B) *Chiloscyllum hasseltii*, (C & D) *C. punctatum*,. (E & F) *Scoliodon laticaudus*, and (G & H) *S. macrorhynchos*.
Dotted line represents 95% confident interval of the regression line.

10.7717/peerj.15849/supp-7 Supplemental Information 7 Reproductive biology sample examined according to species, location and their respective sex ratio (Female: Male), maturity (Im, Immature; Ma, Mature) and total length size range. WP, west coast of Peninsular Malaysia; EP, east coast of Peninsular Malaysia; Borneo, Sarawak and Sabah.

10.7717/peerj.15849/supp-8 Supplemental Information 8 Total, mean and maximum number of prey taxa ingested individually (*N_pi_*) ­for all elasmobranch species and *post-hoc* Kruskal-Wallis ANOVA test among the groups.

10.7717/peerj.15849/supp-9 Supplemental Information 9 Average dissimilarity between shark species (bold) and percentage contribution of the three most important prey items that distinguished their diets based on SIMPER using dietary samples (*%V*, *%N*, *%F* and *%PSIRI*).

10.7717/peerj.15849/supp-10 Supplemental Information 10 Intra- (italic) and inter-specific dietary overlap for sharks using dietary samples %PSIRI.

10.7717/peerj.15849/supp-11 Supplemental Information 11 Raw data obtained from this study separated into Diet, Reproduction, and Embryo.

## Additional Information and Declarations

### Field Study Permissions

The following information was supplied relating to field study approvals (*i.e*., approving body and any reference numbers): Field samplings were approved by the Research Council of the Universiti Malaya (um.tnc1/606/stu2015, um.tnc1/606/stu2016). Collection permits and sampling protocol for specimens form Sabah were approved by the Sabah Biodiversity Council [Access License Reference No: JKM/MBS.1000-2/2 JLD.9 (21–23) and Transfer License Reference No: JKM/MBS. 1000-2/3 JLD.4 (18)].

### Data Availability

The following information was supplied regarding data availability: The raw data are available in the Supplemental Files.

## References

[ref-1] Adams KR, Fetterplace LC, Davis AR, Taylor MD, Knott NA (2018). Sharks, rays and abortion: the prevalence of capture-induced parturition in elasmobranchs. Biological Conservation.

[ref-2] Amariles DF, Navia AF, Giraldo A (2017). Food resource partitioning of the *Mustelus lunulatus* and *Mustelus henlei* (Elasmobranchii: Carcharhiniformes). Environmental Biology of Fishes.

[ref-3] Amundsen PA, Gabler HA, Staldvik FJ (1996). A new approach to graphical analysis of feeding strategy from stomach contents data—modification of the Costello, 1990 method. Journal of Fish Biology.

[ref-4] Anderson MJ (2005). PERMANOVA: a FORTRAN computer program for permutational multivariate analysis of variance.

[ref-5] Arai T, Azri A (2019). Diversity, occurrence and conservation of sharks in the southern South China Sea. PLOS ONE.

[ref-6] Azri A, Arai T (2015). Bycatch and landings of young of the year sharks in the Malaysian South China Sea: implications for conservation. Marine Biodiversity Records.

[ref-7] Bangley CW, Rulifson RA (2017). Habitat partitioning and diurnal nocturnal transition in the elasmobranch community of a North Carolina estuary. Bulletin of Marine Science.

[ref-8] Bleeker P (1852). Bijdrage tot de kennis der Plagiostomen van den Indischen Archipel. Verhandelingen van het Bataviaasch Genootschap van Kunsten en Wetenschappen.

[ref-9] Booth H, Chaya F, Ng S, Tan V, Rao M, Teepol B, Matthews E, Lim A, Gumal M (2021). Elasmobranch fishing and trade in Sarawak, Malaysia, with implications for management. Aquatic Conservation: Marine and Freshwater Ecosystems.

[ref-10] Bornatowski H, Braga RR, Abilhoa V, Corrêa MFM (2014). Feeding ecology and trophic comparisons of six shark species in a coastal ecosystem off southern Brazil. Journal of Fish Biology.

[ref-11] Brown SC, Bizzarro JJ, Cailliet GM, Ebert DA (2012). Breaking with tradition: redefining measures for diet description with a case study of the Aleutian skate *Bathyraja aleutica* (Gilbert 1896). Environmental Biology of Fishes.

[ref-12] Chen W-K, Liu K-M (2006). Reproductive biology of whitespotted bamboo shark *Chiloscyllium plagiosum* in northern waters off Taiwan. Fisheries Science.

[ref-13] Clarke KR, Gorley RN (2006). PRIMER V6, user manual/tutorial.

[ref-14] Cochrane KL (2002). *A fishery manager’s guidebook: management measures and their application*. FAO Fisheries Technical Paper. No. 424.

[ref-15] Compagno LJV (2001). Sharks of the world. An annotated and illustrated catalogue of shark species known to date. Bullhead, mackerel and carpet sharks (Heterodontiformes, Lamniformes and Orectolobiformes).

[ref-16] Conrath CL, Musick JA, Carrier JC, Musick JA, Heithaus MR (2012). Reproductive biology of elasmobranchs. Biology of Sharks and Their Relatives.

[ref-17] Coppock WR, Dziwenka MM, Gupta RC (2017). Endocrine disruption in wildlife species. Reproductive and Developmental Toxicology.

[ref-18] Cortés E (1997). A critical review of methods of studying fish feeding based on analysis of stomach contents: application to elasmobranch fishes. Canadian Journal of Fisheries and Aquatic Sciences.

[ref-19] Cortés E (2000). Life history patterns and correlations in sharks. Reviews in Fisheries Science.

[ref-20] Devadoss P (1979). Observations on the maturity, breeding and development of *Scoliodon laticaudus* muller and henle off Calicut Coast. Journal of the Marine Biological Association of India.

[ref-21] Devadoss P (1989). Observations on the length-weight relationship and food and feeding habits of spadenose shark, *Scoliodon laticaudus* muller and henle. Indian Journal of Fisheries.

[ref-22] Dicken ML, Hussey NE, Christiansen HM, Smale MJ, Nkabi N, Cliff G, Wintner SP (2017). Diet and trophic ecology of the tiger shark (*Galeocerdo cuvier*) from South African waters. PLOS ONE.

[ref-23] DOFM (2006). Malaysia national plan of action for the conservation and management of shark (Putrajaya, 2006). https://www.fao.org/3/br386e/br386e.pdf.

[ref-24] Dudgeon CL, Bennett MB, Kyne PM (2016). *Chiloscyllium punctatum*. The IUCN Red List of Threatened Species 2016: e.T41872A68616745. https://dx.doi.org/10.2305/IUCN.UK.2016-1.RLTS.T41872A68616745.en.

[ref-25] Dulvy NK, Fowler SL, Musick JA, Cavanagh RD, Kyne M, Harrison LR, Carlson JK, Davidson LNK, Sonja V (2014). Extinction risk and conservation of the world’ s sharks and rays. Elife.

[ref-26] Dulvy NK, Simpfendorfer C, Akhilesh KV, Derrick D, Elhassan I, Fernando D, Haque AB, Jabado RW, Maung A, Valinassab T, VanderWright WJ (2021). *Scoliodon laticaudus*. The IUCN Red List of Threatened Species 2021: e.T169234201A173436322. https://dx.doi.org/10.2305/IUCN.UK.2021-2.RLTS.T169234201A173436322.en.

[ref-27] Fahmi F, Oktaviyani S, Bennett MB, Dudgeon CL, Tibbetts R (2021a). Reproductive biology of a bamboo shark as a framework for better fisheries management. Marine and Freshwater Research.

[ref-91] Fahmi TIR, Bennett MB, Ali A, Krajangdara T, Dudgeon CL (2021b). Population structure of the brown-banded bamboo shark *Chiloscyllium punctatum* and its relation to fisheries management in the Indo-Malay region. Fisheries Research.

[ref-28] Ferretti F, Myers RA, Serena F, Lotze HK (2008). Loss of large predatory sharks from the Mediterranean Sea. Conservation Biology.

[ref-29] Ferretti F, Worm B, Britten GL, Heithaus MR, Lotze HK (2010). Patterns and ecosystem consequences of shark declines in the ocean. Ecology Letter.

[ref-30] Fofandi M, Zala MS, Koya M (2013). Observations on selected biological aspects of the spadenose shark (*Scoliodon laticaudus* Müller & Henle, 1838), landed along Saurashtra coast. Indian Journal of Fisheries.

[ref-31] García VB, Lucifora LO, Myers RA (2008). The importance of habitat and life history to extinction risk in sharks, skates, rays and chimaeras. Proceedings of the Royal Society B: Biological Sciences.

[ref-32] Gauthier AR, Whitehead DL, Tibbetts IR, Bennett MB (2019). Comparative morphology of the electrosensory system of the epaulette shark *Hemiscyllium ocellatum* and brown-banded bamboo shark *chiloscyllium punctatum*. Journal of Fish Biology.

[ref-33] Goodwin NB, Dulvy NK, Reynolds JD (2002). Life-history correlates of the evolution of live bearing in fishes. Philosophical Transactions of the Royal Society of London Series B: Biological Sciences.

[ref-34] Gračan R, Zavodnik D, Krstinić P, Dragičević B, Lazar B (2017). Feeding ecology and trophic segregation of two sympatric mesopredatory sharks in the heavily exploited coastal ecosystem of the Adriatic Sea. Journal of Fish Biology.

[ref-35] Gutteridge AN, Huveneers C, Marshall LJ, Tibbetts IR, Bennett MB (2013). Life-history traits of a small-bodied coastal shark. Marine and Freshwater Research.

[ref-36] Harahush BK, Fischer ABP, Collin SP (2007). Captive breeding and embryonic development of *Chiloscyllium punctatum* Muller & Henle, 1838 (Elasmobranchii: Hemiscyllidae). Journal of Fish Biology.

[ref-37] Harahush BK, Hart NS, Collin SP (2014). Ontogenetic changes in retinal ganglion cell distribution and spatial resolving power in the brown-banded bamboo shark *Chiloscyllium punctatum* (Elasmobranchii). Brain, Behaviour and Evolution.

[ref-38] Heithaus MR, Vaudo JJ, Carrier JC, Musick JA, Heithaus MR (2012). Predator-prey interactions. Biology of Sharks and Their Relative.

[ref-39] Heithaus MR, Wirsing AJ, Dill LM (2012). The ecological importance of intact top-predator populations: a synthesis of 15 years of research in a seagrass ecosystem. Marine and Freshwater Research.

[ref-40] Heller R (1980). On optimal diet in a patchy environment. Theoretical Population Biology.

[ref-41] Huveneers C, Ebert DA, Dudley SF (2015). The evolution of chondrichthyan research through a metadata analysis of dedicated international conferences between 1991 and 2014. African Journal of Marine Science.

[ref-42] Hyslop EJ (1980). Stomach contents analysis—a review of methods and their application. Journal of Fish Biology.

[ref-43] Jardas I, Śantić M, Nerlović V, Pallaoro A (2007). Diet of the smooth-hound, *Mustelus mustelus* (Chondrichthyes: Triakidae), in the eastern Adriatic Sea. Cybium.

[ref-44] Kousteni V, Karachle PK, Megalofonou P (2017). Diet and trophic level of the longnose spurdog *Squalus blainville* (Risso, 1826) in the deep waters of the Aegean Sea. Deep Sea Research Part I: Oceanographic Research Papers.

[ref-45] Krebs CJ (1999). Ecological methodology.

[ref-46] Labropoulou M, Eleftheriou A (1997). The foraging ecology of two pairs of congeneric demersal fish species: importance of morphological characteristics in prey selection. Journal of Fish Biology.

[ref-47] Laptikhovsky VV, Arkhipkin AI, Henderson AC (2001). Feeding habits and dietary overlap in spiny dogfish *Squalus acanthias* (Squalidae) and narrowmouth catshark *Schroederichthys bivius* (Scyliorhinidae). Journal of the Marine Biological Association of the United Kingdom.

[ref-48] Last PR, White WT, Caira JN, Dharmadi F, Jensen K, Lim APK, Manjaji-Matsumoto BM, Naylor GJP, Pogonoski JJ, Stevens JD, Yearsley GK (2010). Sharks and Rays of Borneo.

[ref-49] Layman CA, Allgeier JE (2012). Characterizing trophic ecology of generalist consumers: a case study of the invasive lionfish in the Bahamas. Marine Ecology Progress Series.

[ref-50] Levins R (1968). Evolution in changing environment: some theoretical explorations.

[ref-51] Lim KC (2016). Taxonomy of dasyatid stingrays (Myliobatiformes) and a study of their nearshore ecology in Selangor (Malaysia).

[ref-52] Lim KC, Chong VC, Lim P-E, Yurimoto T, Loh KH (2019). Feeding ecology of three sympatric species of stingrays on a tropical mudflat. Journal of the Marine Biological Association of the United Kingdom.

[ref-53] Lim KC, Then AY-H, Wee AKS, Sade A, Rumpet R, Loh K-H (2021). Brown banded bamboo shark (*Chiloscyllium punctatum*) shows high genetic diversity and differentiation in Malaysian waters. Scientific Reports.

[ref-54] Lim KC, White WT, Then AYH, Naylor GJP, Arunrugstichai S, Loh K-H (2022). Integrated taxonomy reveal genetic differences in morphologically similar and non-sympatric *Scoliodon macrorhynchos* and *S. laticaudus*. Animals.

[ref-55] Liu KM, Lin YH, Chang SK, Joung SJ, Su KY (2020). Examining an ontogenetic shift in the diet of the whitespotted bamboo shark *Chiloscyllium plagiosum* in northern Taiwanese waters. Regional Studies in Marine Science.

[ref-56] McKinney ML, Lockwood JL (1999). Biotic homogenization: a few winners replacing many losers in the next mass extinction. Trends in Ecology & Evolution.

[ref-58] Müller J, Henle FGJ (1838). Systematische Beschreibung der Plagiostomen.

[ref-57] Myers RA, Worm B (2005). Extinction, survival or recovery of large predatory fishes. Philosophical Transactions of the Royal Society B.

[ref-59] Navia AF, Mejía-Falla PA, Giraldo A (2007). Feeding ecology of elasmobranch fishes in coastal waters of the Colombian Eastern Tropical Pacific. BMC Ecology.

[ref-60] Nur-Farhana A, Samat A, Zaidi CC, Mazlan AG (2013). Stomach content and trophic level position of two bamboo shark species *Chiloscyllium indicum* and *C. hasseltii* (Hemiscylliidae) from south eastern waters of Peninsular Malaysia. Journal of Sustainability Science and Management.

[ref-61] Osmany HB, Manzoor H, Zohra K (2018). Stomach content analysis of spadenose shark (*Scoliodon laticaudus*) Muller & Henle, 1838 from the coast of Pakistan. International Journal of Biology and Biotechnology.

[ref-62] O’Shea OR, Thums M, van Keulen M, Kempster RM, Meekan MG (2013). Dietary partitioning by five sympatric species of stingray (Dasyatidae) on coral reefs. Journal of Fish Biology.

[ref-63] Pardo SA, Burgess KB, Teixeira D, Bennett MB (2015). Local-scale resource partitioning by stingrays on an intertidal flat. Marine Ecology Progress Series.

[ref-64] Parsons GR, Hoffmayer ER, Frank J, Bet-Sayad W, Rocha MJ, Arukwe A, Kapoor BG (2008). A review of shark reproductive ecology: life history and evolutionary implications. Fish Reproduction.

[ref-65] Platell ME, Potter IC (2001). Partitioning of food resources amongst 18 abundant benthic carnivorous fish species in marine waters on the lower west coast of Australia. Journal of Experimental Marine Biology and Ecology.

[ref-66] Rigby CL, Bin Ali A, Chen X, Derrick D, Dharmadi Ebert, Fahmi DA, Fernando D, Gautama DA, Ho H, Hsu H, Krajangdara T, Maung A, Sianipar A, Tanay D, Utzurrum JAT, Vo VQ, Yuneni RR, Zhang J (2020). *Scoliodon macrorhynchos*. The IUCN Red List of Threatened Species 2020: e.T169233669A169233911. https://dx.doi.org/10.2305/IUCN.UK.2020-3.RLTS.T169233669A169233911.en.

[ref-67] Rosende-Pereiro A, Flores-Ortega JR, González-Sansón G, Corgos A (2020). Stomach content and stable isotopes reveal an ontogenetic dietary shift of young-of-the-year scalloped hammerhead sharks (*Sphyrna lewini*) inhabiting coastal nursery areas. Environmental Biology of Fishes.

[ref-68] Ruocco NL, Lucifora LO (2016). Ecological singularity of temperate mesopredatory myliobatid rays (Chondrichthyes: Myliobatiformes). Marine and Freshwater Research.

[ref-77] Sánchez-Hernández J, Gabler H-M, Amundsen P-A (2017). Prey diversity as a driver of resource partitioning between river-dwelling fish species. Ecology and Evolution.

[ref-69] Saïdi B, Enajjar S, Bradai MN, Bouain A (2009). Diet composition of smooth-hound shark, *Mustelus mustelus* (Linnaeus, 1758), in the Gulf of Gabès, southern Tunisia. Journal of Applied Ichthyology.

[ref-70] Schindler DE, Essington TE, Kitchell JF, Boggs C, Hilborn R (2002). Sharks and tunas: fisheries impacts on predators with contrasting life histories. Ecological Applications.

[ref-71] Scholz C, Firozpoor J, Kramer-Schadt S, Gras P, Schulze C, Kimmig SE, Woigt CC, Ortmann S (2020). Individual dietary specialization in a generalist predator: a stable isotope analysis of urban and rural red foxes. Ecology and Evolution.

[ref-72] Sen S, Chakraborty SK, Zacharia PU, Dash G, Kizhakudan SJ, Bharadiya SA, Gohel JK (2018). Reproductive strategy of spadenose shark, *Scoliodon laticaudus* Muller and Henle, 1839 along north-eastern Arabian Sea. Journal of Applied Ichthyology.

[ref-73] Sommerville E, Platell ME, White WT, Jones AA, Potter IC (2011). Partitioning of food resources by four abundant, co-occurring elasmobranch species: relationships between diet and both body size and season. Marine and Freshwater Research.

[ref-74] StatSoft Inc (1997). Electronic statistics textbook.

[ref-75] Stergiou KI (2002). Overfishing, tropicalization of fish stocks, uncertainty and ecosystem management: resharpening Ockham’s razor. Fisheries Research.

[ref-76] Stevens JD, Bonfil R, Dulvy NK, Walker PA (2000). The effect of fishing on sharks, rays and chimaeras (Chondrichthyans) and the implications for marine ecosystems. ICES Journal of Marine Science.

[ref-78] Teshima K, Ahmad M, Mizue K (1978). Studies on sharks-XIV. Reproduction in the Telok Anson shark collected from Perak River, Malaysia. Japanese Journal of Ichthyology.

[ref-79] Torres-Rojas YE, Páez Osuna F, Camalich J, Galván Magaña F (2015). Diet and trophic level of scalloped hammerhead shark (*Sphyrna lewini*) from the Gulf of California and Gulf of Tehuantepec, Mexico. Iranian Journal of Fisheries Sciences.

[ref-80] Treloar MA, Laurenson LJB, Stevens JD (2007). Dietary comparisons of six skate species (Rajidae) in south-eastern Australian waters. Environmental Biology of Fishes.

[ref-81] Troudet J, Grandcolas P, Blin A, Vignes-Lebbe R, Legendre F (2017). Taxonomic bias in biodiversity data and societal preferences. Scientific Reports.

[ref-82] Valladares F, Bastias CC, Godoy O, Granda E, Escudero A (2015). Species coexistence in a changing world. Frontiers in Plant Science.

[ref-83] VanderWright WJ, Bin Ali A, Derrick D, Dharmadi F, Haque AB, Krajangdara T, Maung A, Seyha L, Vo VQ, Yuneni RR (2020). *Chiloscyllium hasseltii*. The IUCN Red List of Threatened Species 2020: e.T161557A124506268. https://dx.doi.org/10.2305/IUCN.UK.2020-3.RLTS.T161557A124506268.en.

[ref-84] Wang J, Qiu S, He Y, Yang S, Liu X, Chen M (1996). Feeding habits of spadenose shark, *Scoliodon laticaudus* from southern coast of Fujian. Journal of Oceanography in Taiwan Strait.

[ref-85] White WT (2007). Aspects of the biology of carcharhiniform sharks in Indonesian waters. Journal of the Marine Biological Association of the United Kingdom.

[ref-86] White WT, Platell ME, Potter IC (2001). Relationship between reproductive biology and age composition and growth in *Urolophus lobatus* (Batoidea: Urolophidae). Marine Biology.

[ref-87] Wilga CAD, Lauder GV, Jeffrey CC, Musick JA, Heithaus MR (2004). Biomechanics of locomotion in sharks, rays, and chimeras. Biology of Sharks and their Relatives.

[ref-88] Woo KJ, Elliott KH, Davidson M, Gaston AJ, Davoren GK (2008). Individual specialization in diet by a generalist marine predator reflects specialization in foraging behaviour. Journal of Animal Ecology.

[ref-89] Woodland RJ, Secor DH, Wedge ME (2011). Trophic resource overlap between small elasmobranchs and sympathric teleosts in Mid-Atlantic Bight nearshore habitats. Estuaries and Coasts.

[ref-90] Zhao Y, Jiang C, Ju P, Xiao J, Chen M (2022). Reproductive biology of the Pacific spadenose shark *Scoliodon macrorhynchos*, a heavily exploited species in the Southern Taiwan Strait. Marine and Coastal Fisheries.

