# Supplementary figures and images for: Feeding ecology and reproductive biology of small coastal sharks in Malaysian waters
Source: PeerJ. 2023 Aug 21;11:e15849. doi: 10.7717/peerj.15849 (PMC10448880; doi:10.7717/peerj.15849)

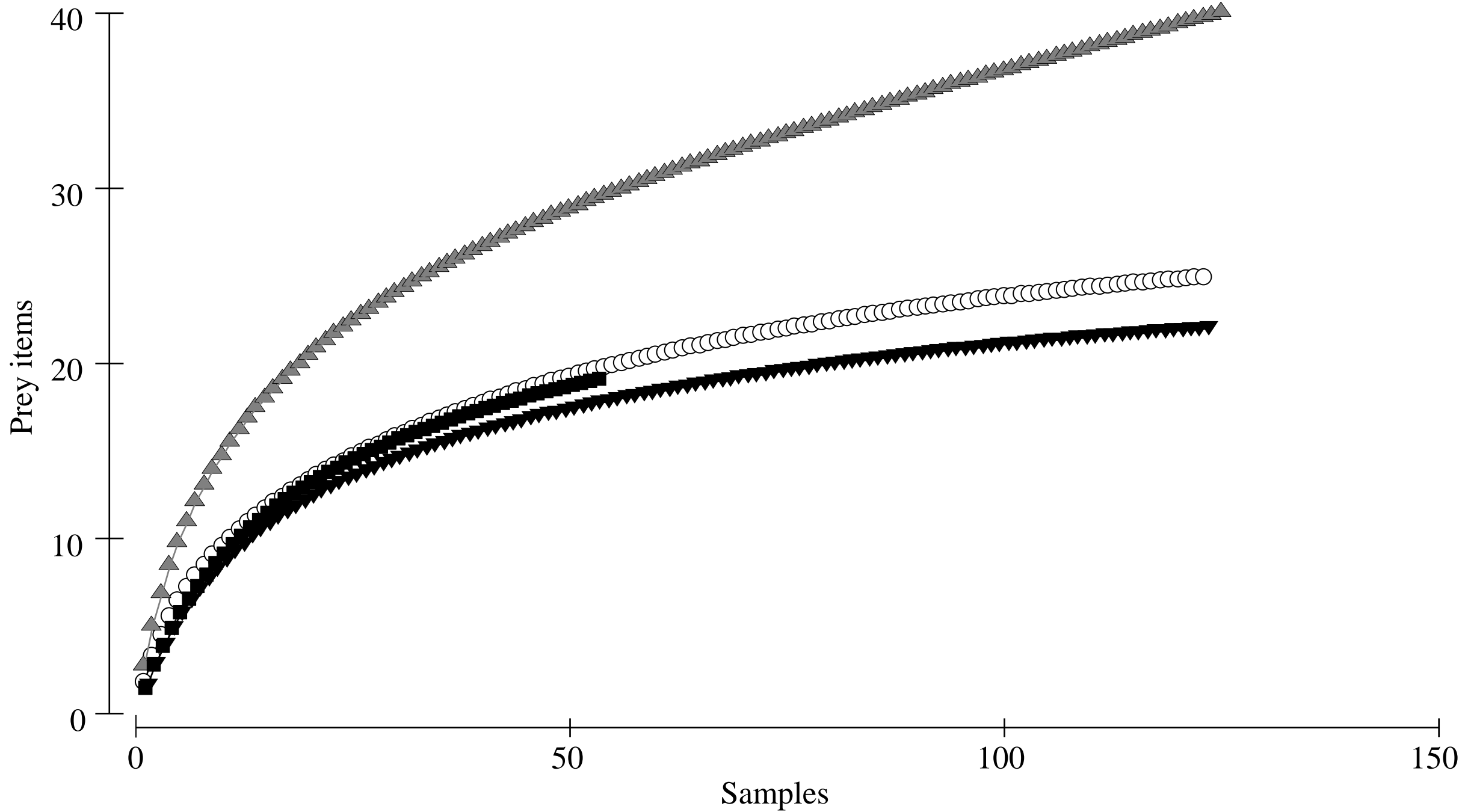

Supplement: Supplemental Information 1 — Grey upward triangle, C. hasseltii; black downward triangle, C. punctatum; empty circle, S. laticaudus; black square, S. macrorhynchos. [file peerj-11-15849-s001.pdf]

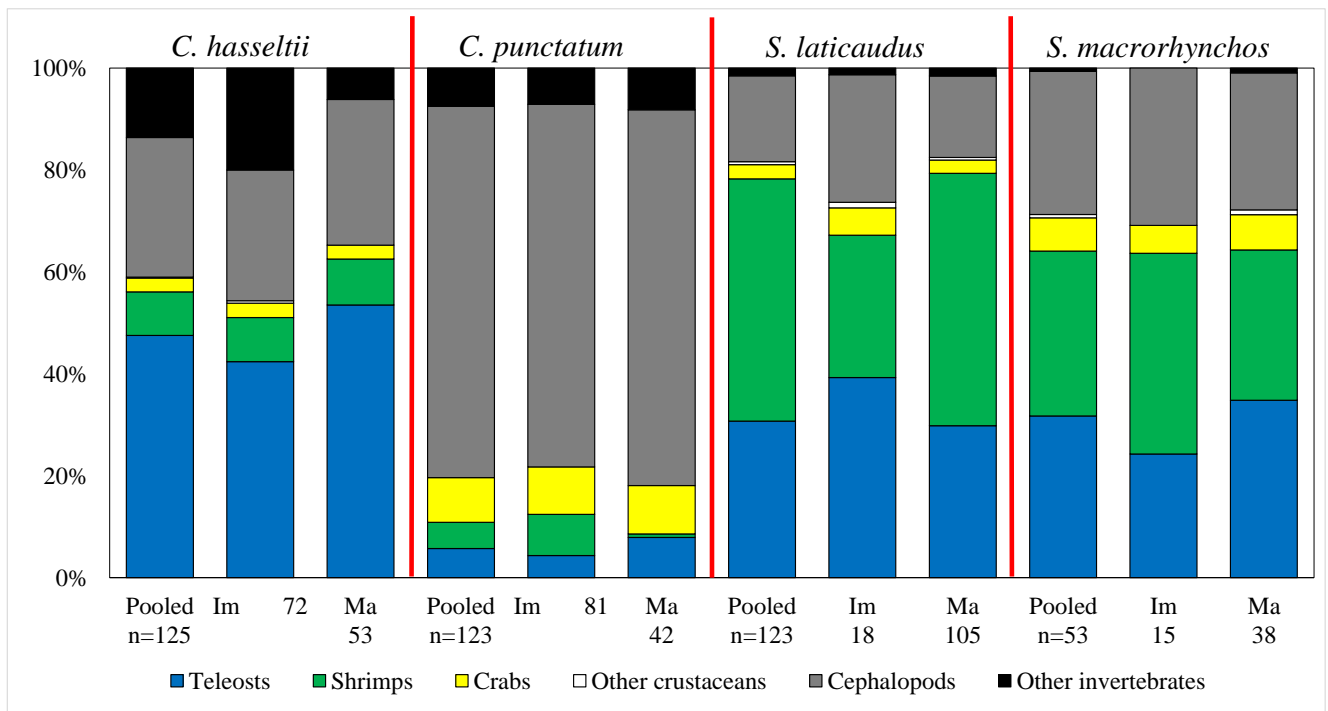

Supplement: Supplemental Information 2 — Im, immature; Ma, mature. [file peerj-11-15849-s002.pdf]

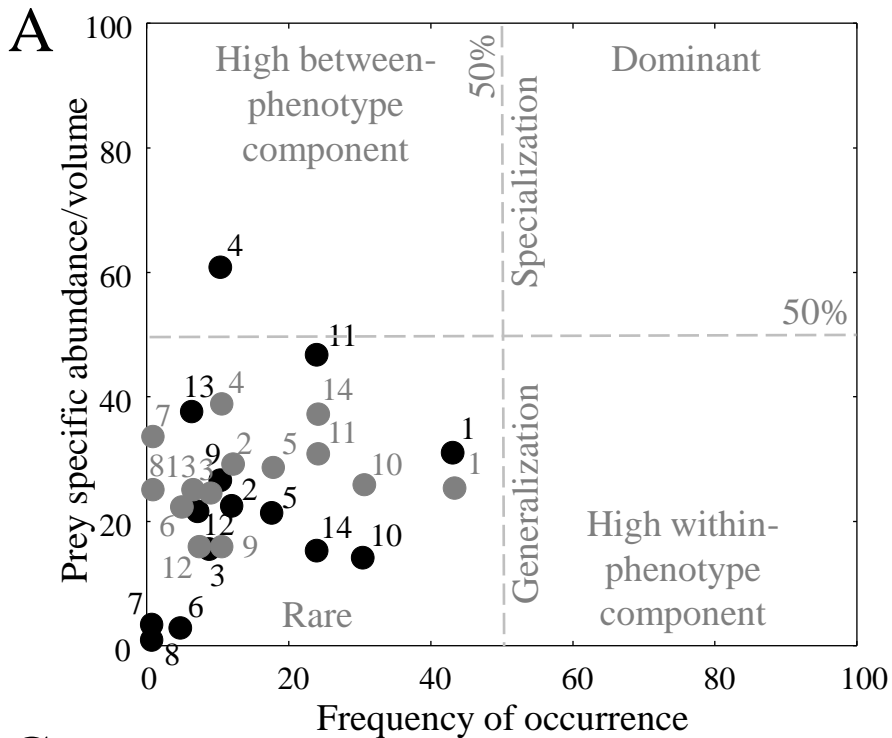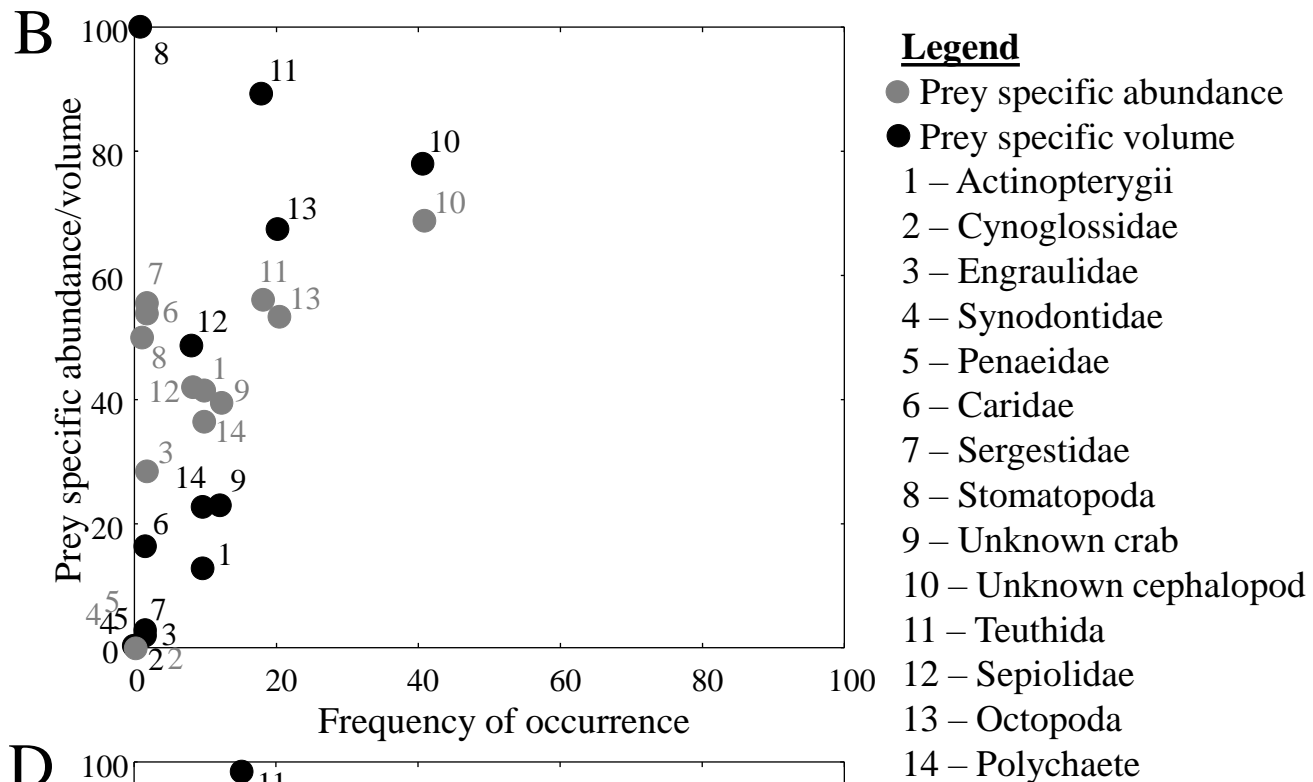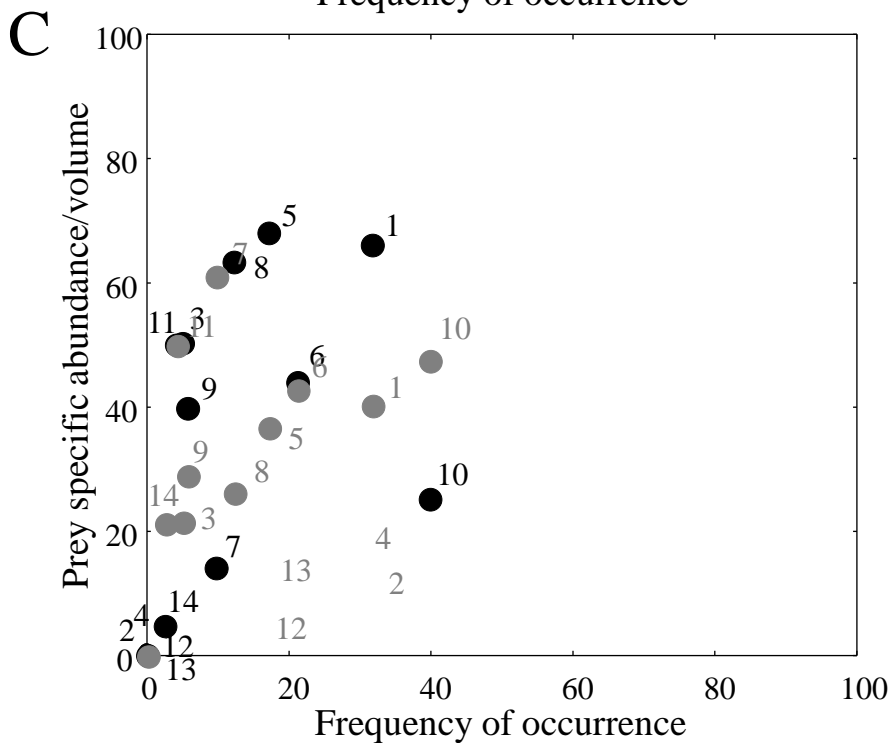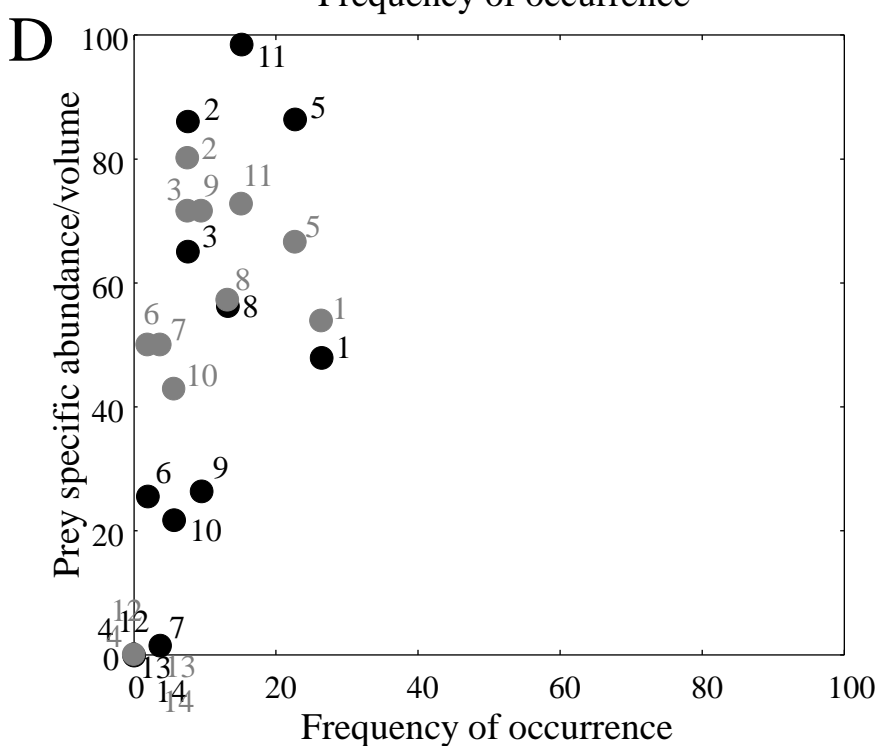

Supplement: Supplemental Information 3 — (A) C. hasseltii, (B) C. punctatum, (C) S. laticaudus, and (D) S. macrorhynchos. Grey symbols, prey specific abundance; black symbols, prey specific volume. [file peerj-11-15849-s003.pdf]

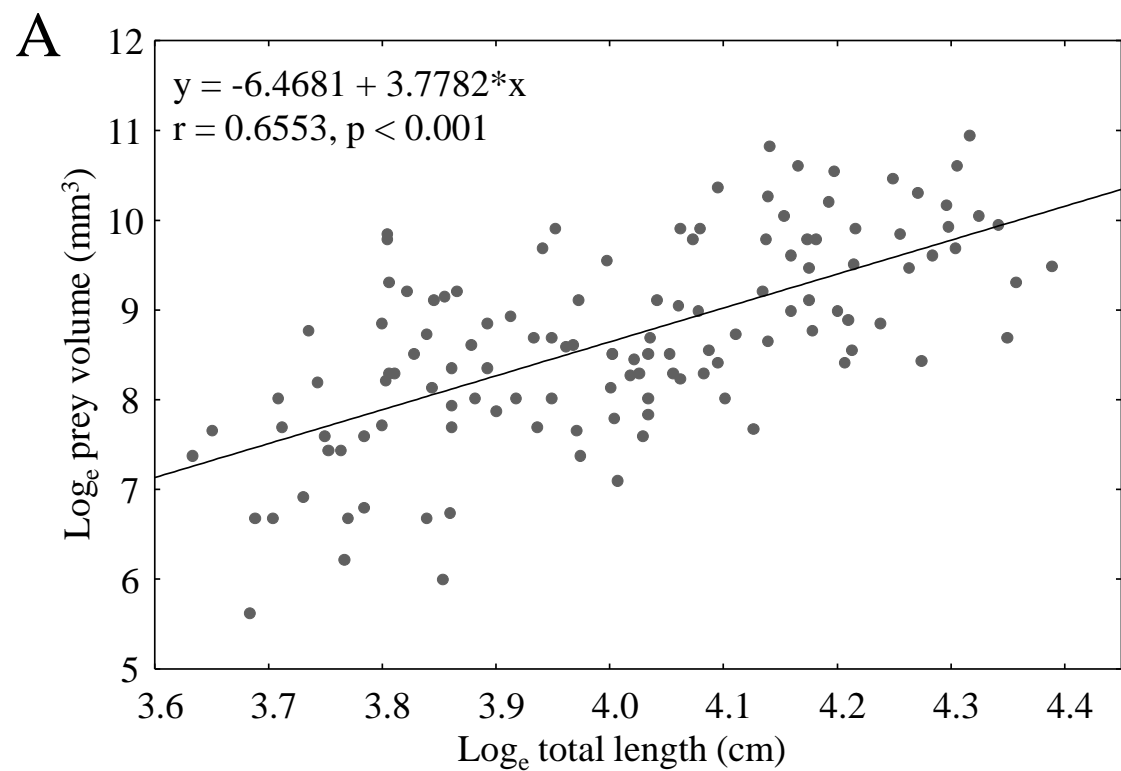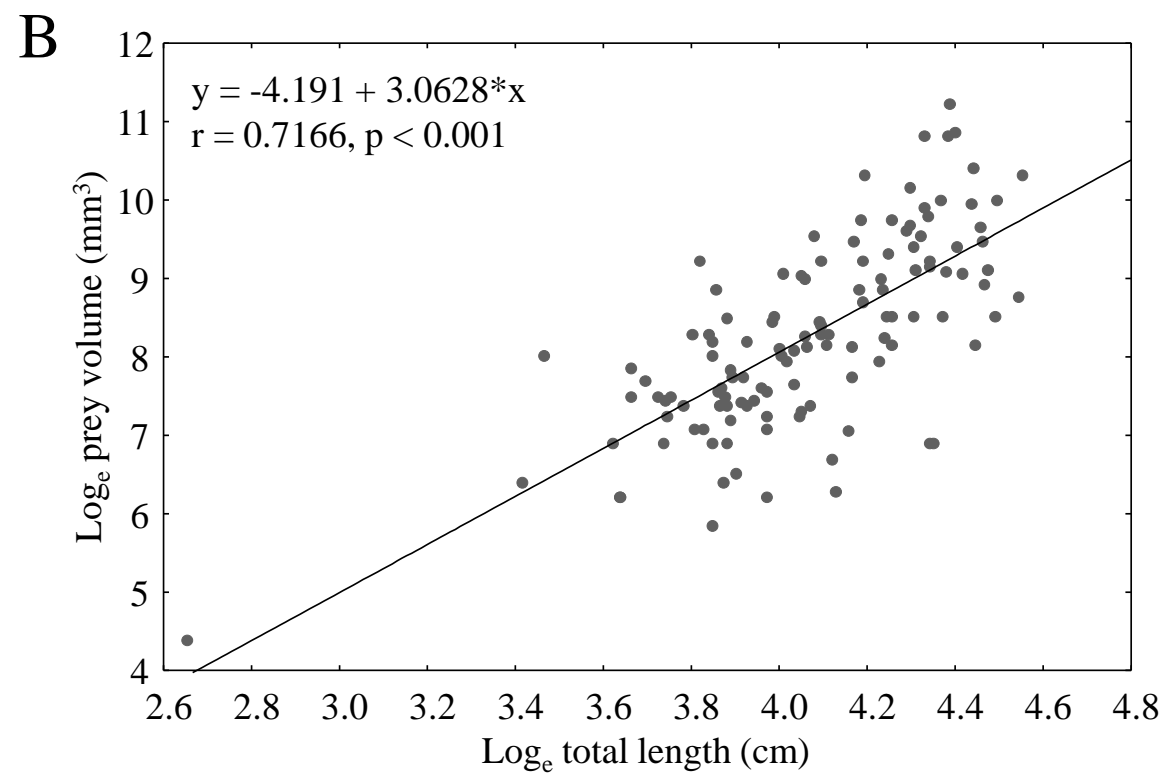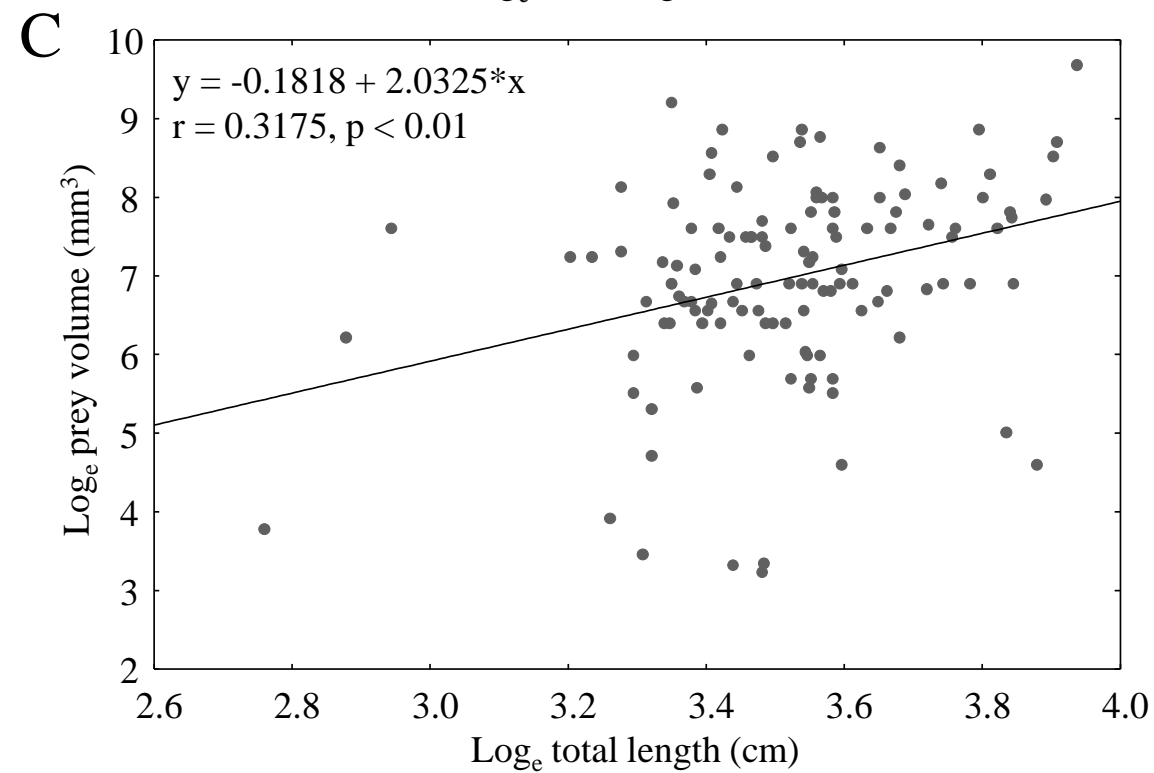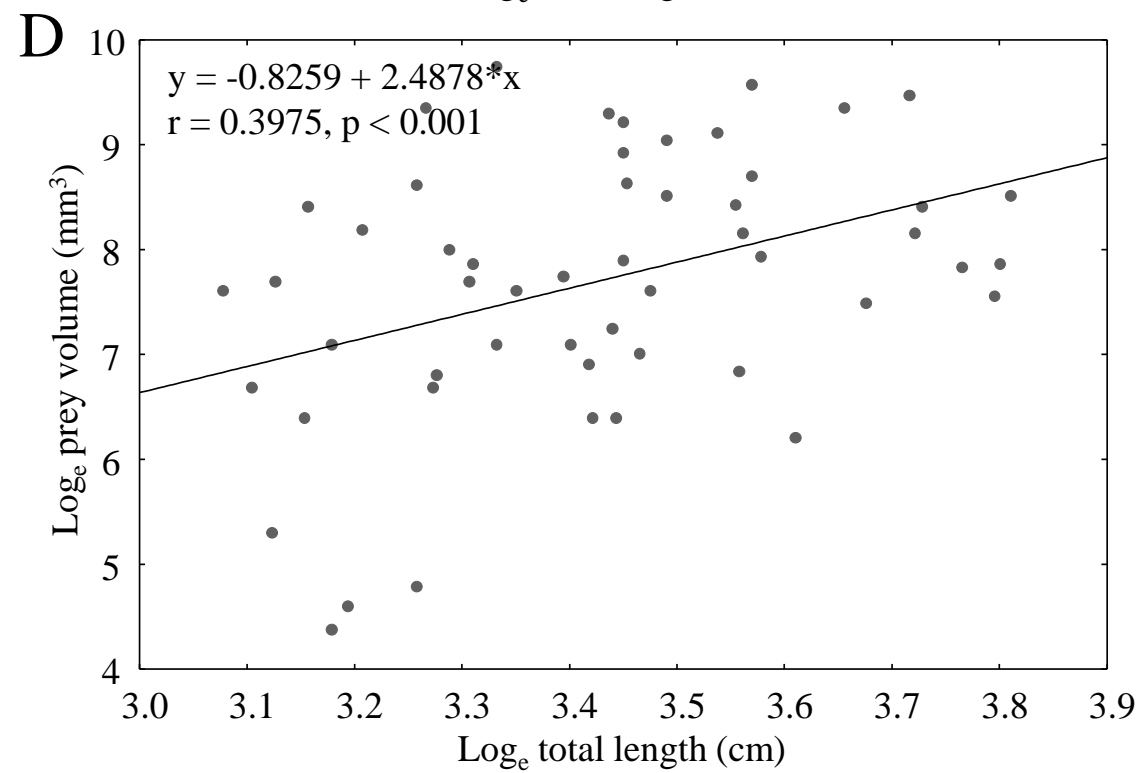

Supplement: Supplemental Information 4 — (A) Chiloscyllium hasseltii, (B) C. punctatum, (C) S. laticaudus, and (D) S. macrorhynchos. [file peerj-11-15849-s004.pdf]

**A**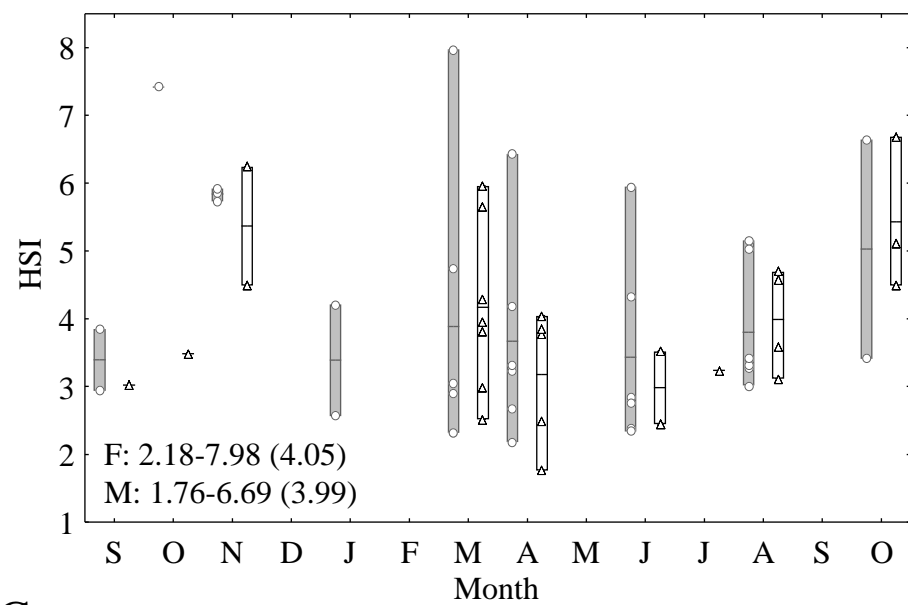**B**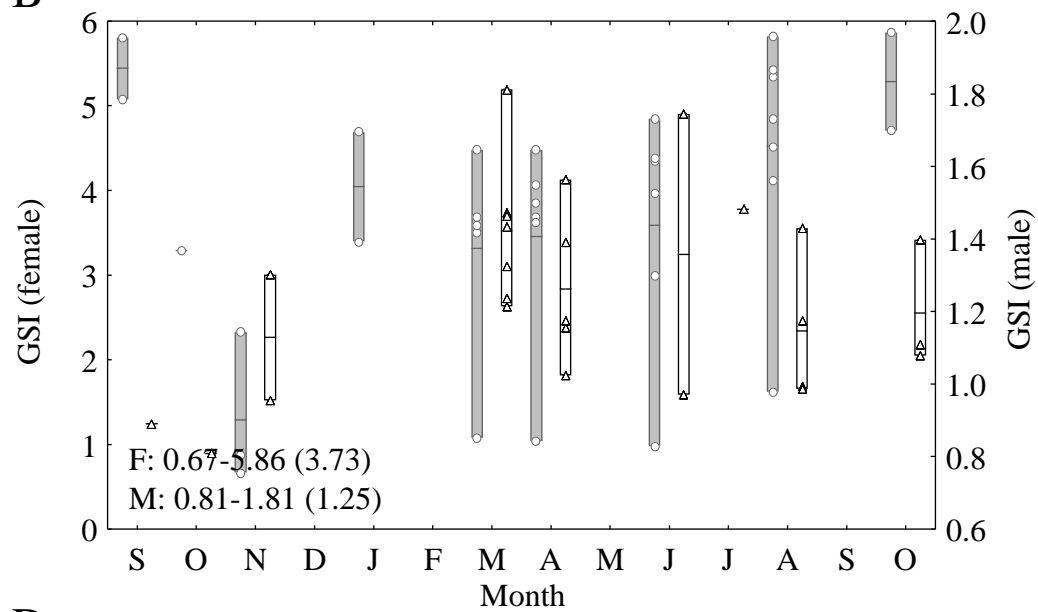**C**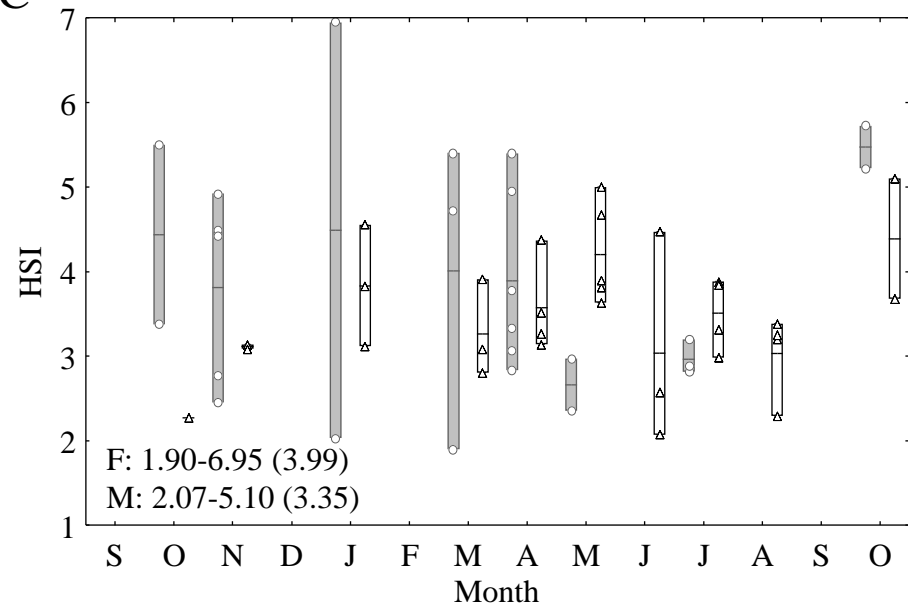**D**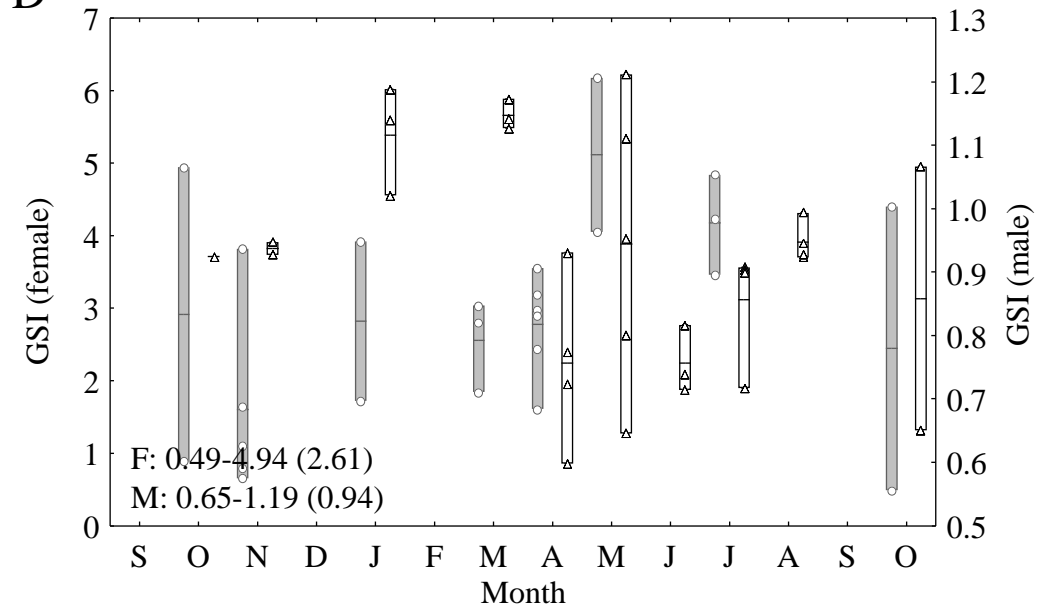**E**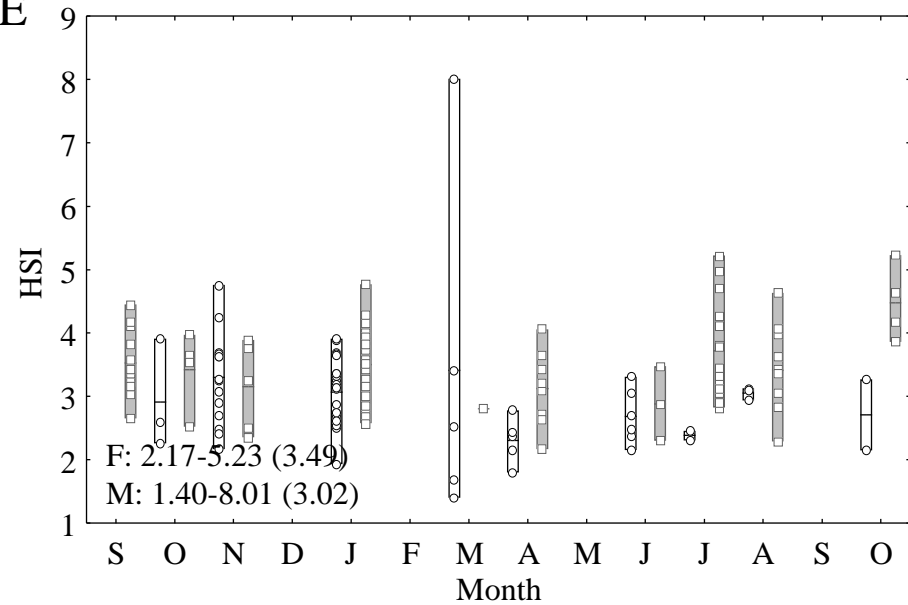**F**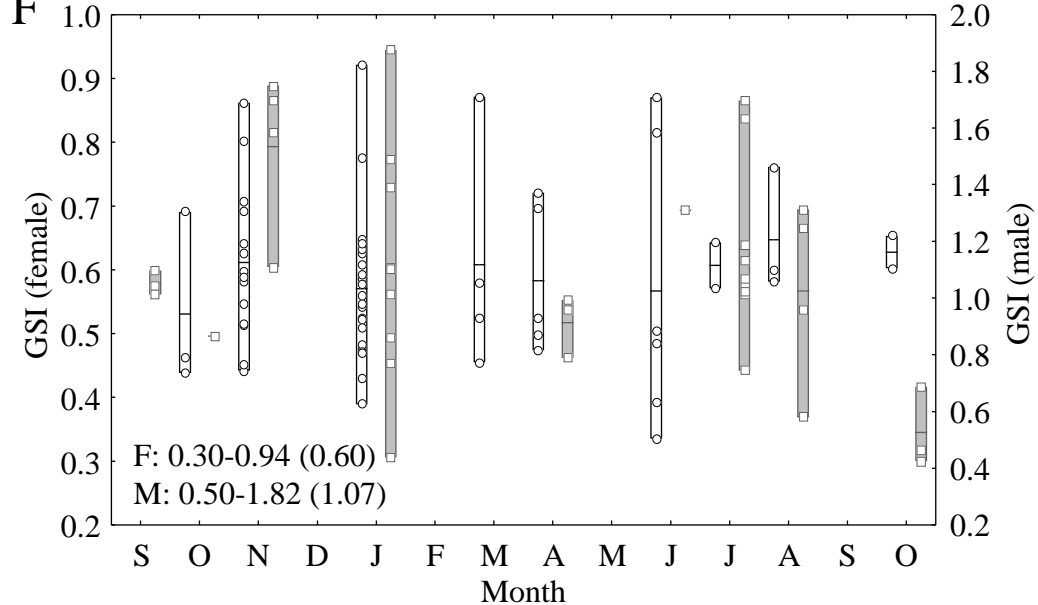

Supplement: Supplemental Information 5 — Grey filled box, female (F); Unshaded box, male (M). Range and mean (in parenthesis) were stated on the lower left corner. [file peerj-11-15849-s005.pdf]

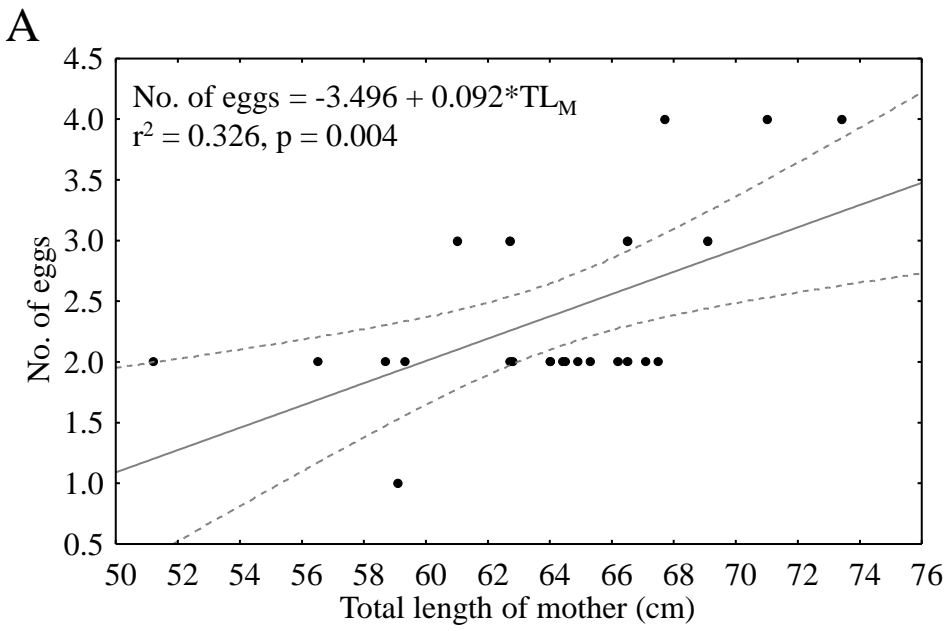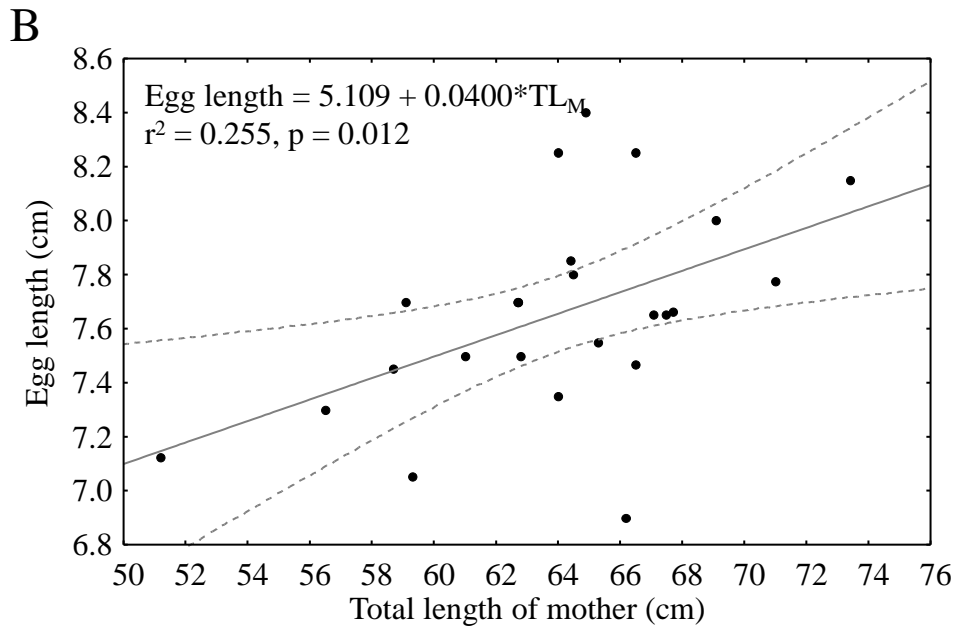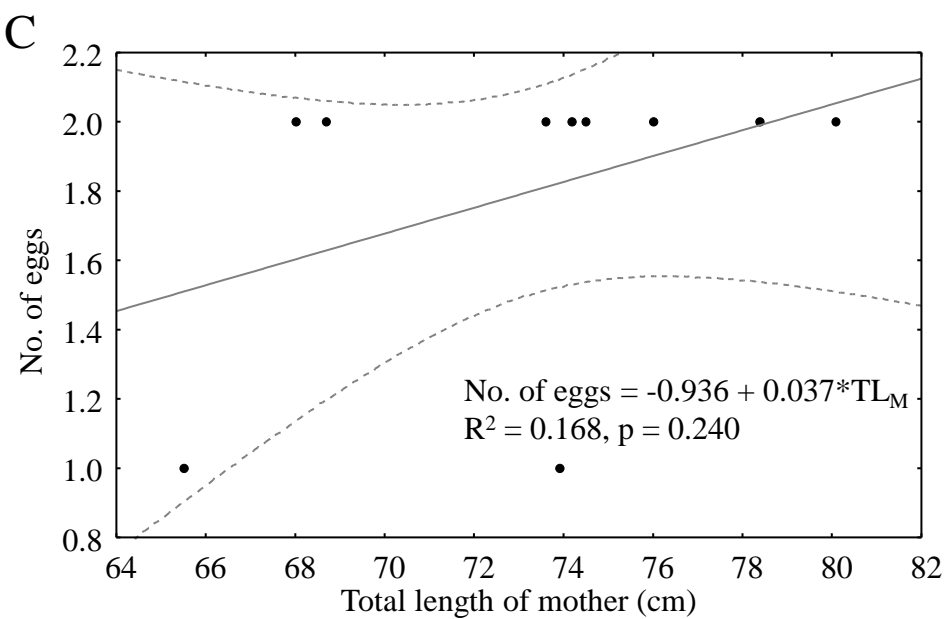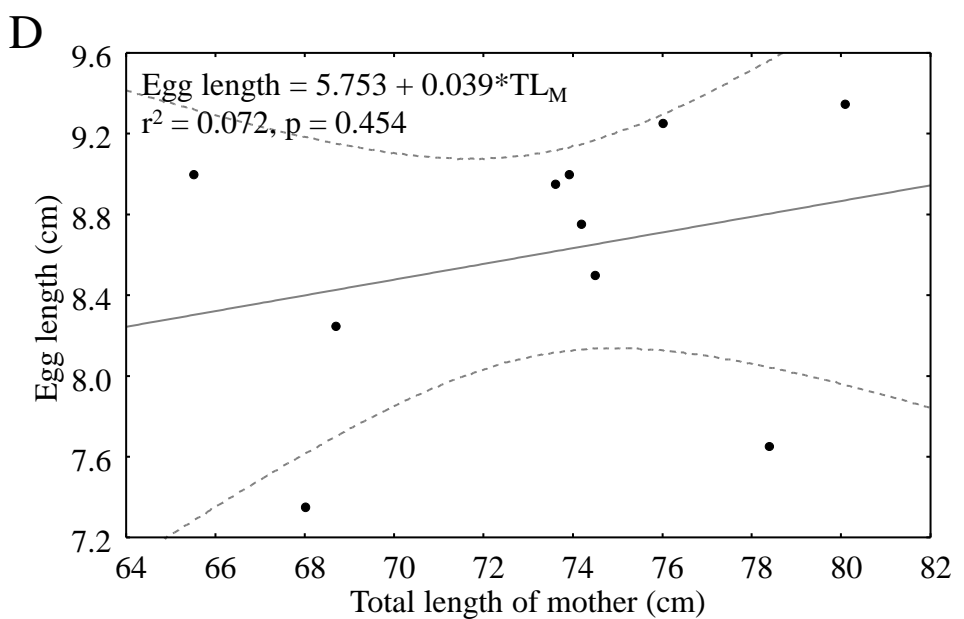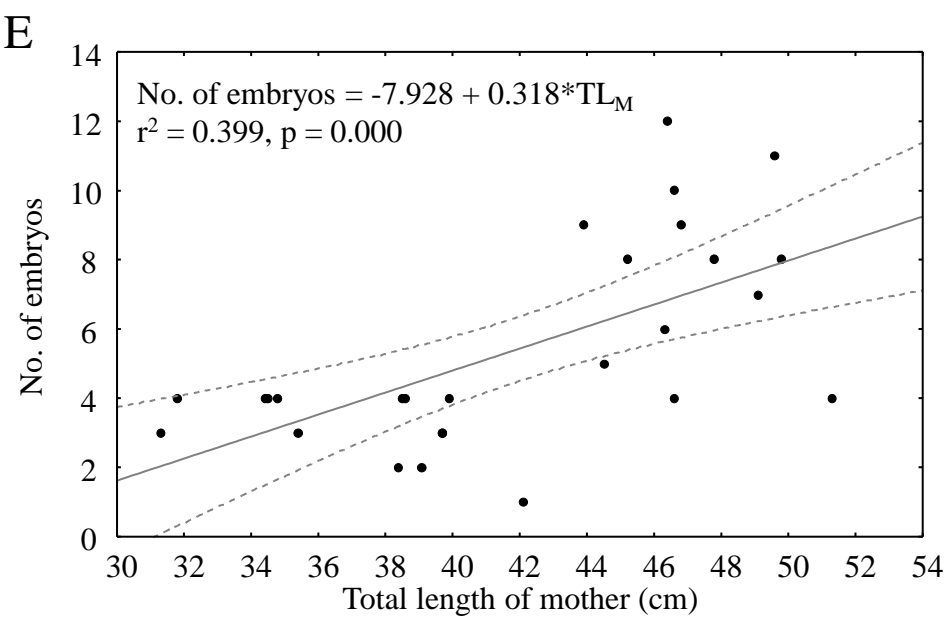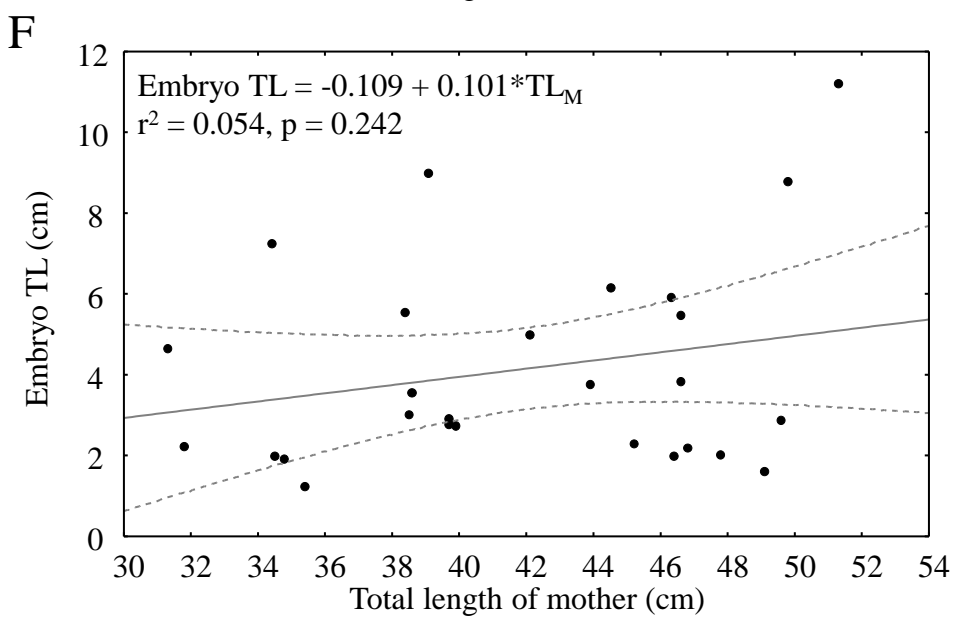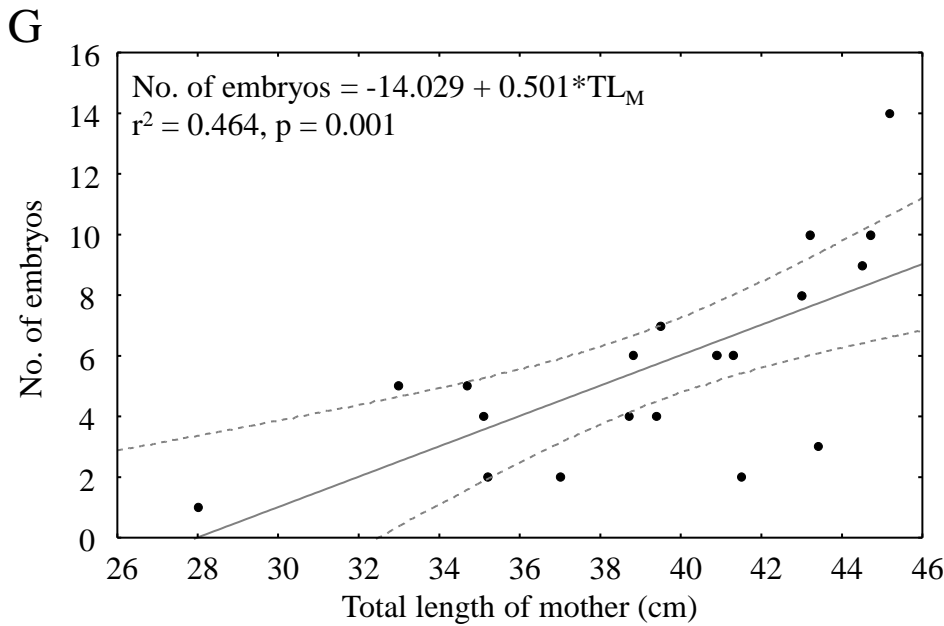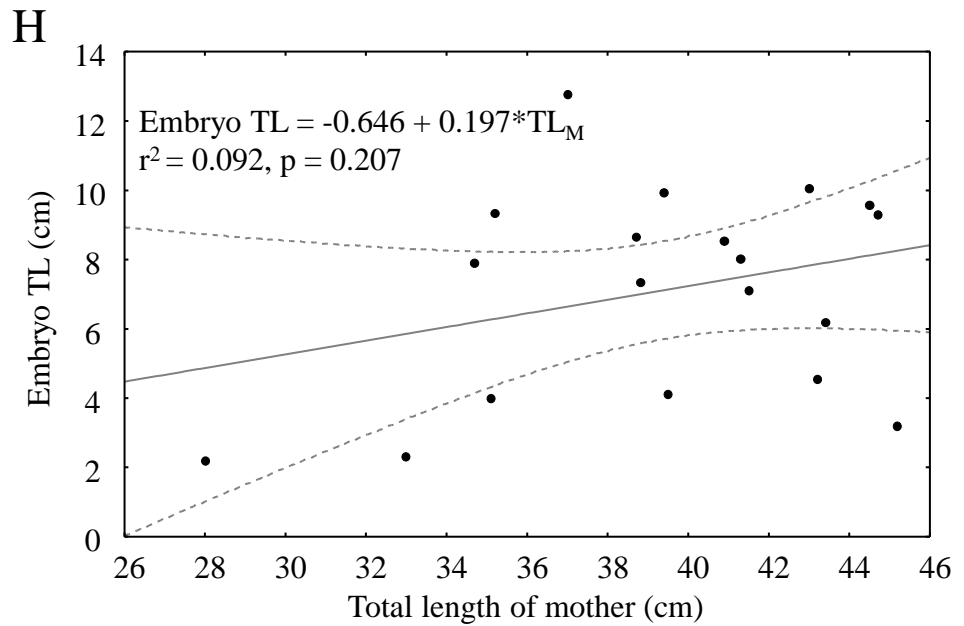

Supplement: Supplemental Information 6 — (E & F) Scoliodon laticaudus, and (G & H) S. macrorhynchos. Dotted line represents 95% confident interval of the regression line. [file peerj-11-15849-s006.pdf]
