# Supplementary material for: Feeding ecology and reproductive biology of small coastal sharks in Malaysian waters
Source: PeerJ. 2023 Aug 21;11:e15849. doi: 10.7717/peerj.15849 (PMC10448880; doi:10.7717/peerj.15849)
Supplement: Supplemental Information 7 — WP, west coast of Peninsular Malaysia; EP, east coast of Peninsular Malaysia; Borneo, Sarawak and Sabah. [file peerj-11-15849-s007.docx]

**Table 2: Reproductive biology sample examined according to species, location and their respective sex ratio (Female: Male), maturity (Im = Immature, Ma = Mature) and total length size range**. WP = west coast of Peninsular Malaysia. EP = east coast of Peninsular Malaysia, Borneo = Sarawak and Sabah.

| **Species** | **Location** |  |  | **Maturity** | | **Size range (cm)** | | |
| --- | --- | --- | --- | --- | --- | --- | --- | --- |
|  |  | **Total** | **Sex ratio (F:M)** | **Im** | **Ma** | **Min** | **Max** | **Mean** |
| *C. hasseltii* | WP | 181 | 1:1.8 | 101 | 80 | 37.8 | 80.6 | 57.4 |
|  | EP | 11 | 1:2.7 | 5 | 6 | 45.0 | 71.0 | 57.9 |
|  | Sarawak | 2 | 2:0 |  | 2 | 61.0 | 64.5 | 62.8 |
|  | All | 194 | 1:1.8 | 106 | 88 | 37.8 | 80.6 | 57.5 |
| *C. punctatum* | WP | 165 | 1:1.5 | 111 | 54 | 30.5 | 102.0 | 62.5 |
|  | EP | 153 | 1:2.4 | 59 | 94 | 14.2 | 97.5 | 69.5 |
|  | Borneo | 40 | 1:5.7 | 14 | 26 | 46.0 | 89.0 | 71.3 |
|  | All | 358 | 1:2.1 | 184 | 174 | 14.2 | 102.0 | 66.5 |
| *S. laticaudus* | WP | 178 | 1:0.9 | 23 | 155 | 11.4 | 51.3 | 35.0 |
| *S. macrorhynchos* | Sarawak | 128 | 1:1.4 | 31 | 97 | 20.9 | 49.5 | 33.2 |
