# Supplementary material for: Feeding ecology and reproductive biology of small coastal sharks in Malaysian waters
Source: PeerJ. 2023 Aug 21;11:e15849. doi: 10.7717/peerj.15849 (PMC10448880; doi:10.7717/peerj.15849)
Supplement: Supplemental Information 8 [file peerj-11-15849-s008.docx]

**Table S2: Total, mean and maximum number of prey taxa ingested individually (*N_pi_*) ­for all elasmobranch species and post-hoc Kruskal-Wallis ANOVA test among the groups.**

| **Group** | **Total N_pi_** | **Mean N_pi_** | **Max N_pi_** | **Chas** | **Cpun** | **Slat** | **Smac** |
| --- | --- | --- | --- | --- | --- | --- | --- |
| All | 45 | 2.0 | 7 |  |  |  |  |
| Chas | 39 | 2.8 | 7 | - | 0.000 | 0.000 | 0.000 |
| Cpun | 22 | 1.6 | 5 | 7.194* | - | 0.186 | 1.000 |
| Slat | 25 | 1.9 | 5 | 5.027* | 2.157 | - | 0.107 |
| Smac | 19 | 1.4 | 3 | 6.270* | 0.695 | 2.369 | - |

*indicate significant difference at P < 0.05; Lower and upper triangular (shaded) refers to z-values and P values for the test respectively. Chas = *C. hasseltii*, Cpun = *C. punctatum*, Slat = *S. laticaudus*, Smac = *S. macrorhynchos*
