# Supplementary material for: Feeding ecology and reproductive biology of small coastal sharks in Malaysian waters
Source: PeerJ. 2023 Aug 21;11:e15849. doi: 10.7717/peerj.15849 (PMC10448880; doi:10.7717/peerj.15849)
Supplement: Supplemental Information 9 [file peerj-11-15849-s009.docx]

**Table 5:** **Average dissimilarity between shark species (bold) and percentage contribution of the three most important prey items that distinguished their diets based on SIMPER using dietary samples (*%V*, *%N*, *%F* and *%PSIRI*).**

|  | ***%V*** | ***%N*** | ***%F*** | ***%PSIRI*** |
| --- | --- | --- | --- | --- |
| Chas x Cpun | **78.57** | **75.27** | **68.99** | **75.82** |
| 1 | Teuth 16.3 | Cepha 14.5 | Actin 11.7 | Teuth 12.8 |
| 2 | Octop 14.8 | Teuth 9.6 | Cepha 8.5 | Cepha 12.4 |
| 3 | Cepha 10.3 | Nemat 7.2 | Polyc 7.3 | Octop 10.9 |
| Chas x Slat | **83.07** | **75.60** | **66.96** | **77.41** |
| 1 | Teuth 11.8 | Cepha 12.3 | Actin 9.2 | Actin 10.4 |
| 2 | Actin 11.2 | Actin 10.4 | Polyc 7.5 | Teuth 9.2 |
| 3 | Penae 9.1 | Carid 6.9 | Cepha 7.5 | Cepha 8.1 |
| Chas x Smac | **75.81** | **74.95** | **71.14** | **73.52** |
| 1 | Teuth 21.0 | Penae 10.0 | Actin 9.3 | Teuth 14.7 |
| 2 | Penae 15.1 | Teuth 8.5 | Cepha 9.12 | Penae 12.3 |
| 3 | Actin 8.4 | Actin 7.2 | Polyc 8.0 | Actin 7.0 |
| Cpun x Slat | **88.25** | **74.11** | **70.83** | **79.81** |
| 1 | Teuth 15.4 | Cepha 12.9 | Actin 11.5 | Teuth 12.6 |
| 2 | Octop 14.2 | Actin 11.0 | Cepha 9.6 | Octop 11.4 |
| 3 | Actin 10.2 | Teuth 9.0 | Carid 8.5 | Actin 10.6 |
| Cpun x Smac | **80.97** | **83.20** | **80.94** | **81.05** |
| 1 | Teuth 20.5 | Cepha 15.4 | Cepha 15.8 | Teuth 14.4 |
| 2 | Penae 15.6 | Penae 10.5 | Penae 10.4 | Penae 13.2 |
| 3 | Octop 15.5 | Teuth 8.9 | Octop 8.4 | Cepha 13.0 |
| Slat x Smac | **79.22** | **74.96** | **70.55** | **74.31** |
| 1 | Teuth 21.3 | Cepha 15.4 | Cepha 16.4 | Teuth 15.0 |
| 2 | Penae 15.3 | Actin 11.3 | Actin 11.6 | Penae 11.9 |
| 3 | Actin 10.6 | Penae 9.3 | Carid 9.1 | Actin 10.5 |

Chas = *C. hasseltii*, Cpun = *C. punctatum*, Slat = *S. laticaudus,* Smac = *S. macrorhynchos.* Prey abbreviations see Table 3.
