# Supplementary material for: Feeding ecology and reproductive biology of small coastal sharks in Malaysian waters
Source: PeerJ. 2023 Aug 21;11:e15849. doi: 10.7717/peerj.15849 (PMC10448880; doi:10.7717/peerj.15849)
Supplement: Supplemental Information 10 [file peerj-11-15849-s010.docx]

**Table S4: Intra- (italic) and inter-specific dietary overlap for sharks using dietary samples %PSIRI.**

|  | *C. hasseltii* | *C. punctatum* | *S. laticaudus* | *S. macrorhynchos* |
| --- | --- | --- | --- | --- |
| *C. hasseltii* | *35.72* | - | - | - |
| *C. punctatum* | 24.18 | *40.92* | - | - |
| *S. laticaudus* | 22.59 | 20.19 | *35.52* | - |
| *S. macrorhynchos* | 26.48 | 18.95 | 25.69 | *34.64* |
